# Robust and consistent biomarker candidates identification by a machine learning approach applied to pancreatic ductal adenocarcinoma metastasis

**DOI:** 10.1186/s12911-024-02578-0

**Published:** 2024-06-20

**Authors:** Tanakamol Mahawan, Teifion Luckett, Ainhoa Mielgo Iza, Natapol Pornputtapong, Eva Caamaño Gutiérrez

**Affiliations:** 1 Program in Bioinformatics and Computational Biology, Graduate School, Chulalongkorn University, Bangkok, Thailand; 2 Department of Biochemistry & System Biology, Institute of Systems, Molecular and Integrative Biology, University of Liverpool, Liverpool, UK; 3 Akkhraratchakumari Veterinary College, Walailak University, Nakhon Si Thammarat, Thailand; 4 Department of Molecular and Clinical Cancer Medicine, Institute of Systems, Molecular and Integrative Biology, University of Liverpool, Liverpool, UK; 5 Department of Biochemistry and Microbiology, Faculty of Pharmaceutical Sciences, and Center of Excellence in Systems Biology, Faculty of Medicine, Chulalongkorn University, Bangkok, Thailand; 6 Computational Biology Facility, LIV-SRF, Faculty of Health and Life Sciences, University of Liverpool, Liverpool, UK

**Keywords:** Biomarker identification, Machine Learning, PDAC, Pancreatic cancer, Metastasis

## Abstract

**Background:**

Machine Learning (ML) plays a crucial role in biomedical research. Nevertheless, it still has limitations in data integration and irreproducibility. To address these challenges, robust methods are needed. Pancreatic ductal adenocarcinoma (PDAC), a highly aggressive cancer with low early detection rates and survival rates, is used as a case study. PDAC lacks reliable diagnostic biomarkers, especially metastatic biomarkers, which remains an unmet need. In this study, we propose an ML-based approach for discovering disease biomarkers, apply it to the identification of a PDAC metastatic composite biomarker candidate, and demonstrate the advantages of harnessing data resources.

**Methods:**

We utilised primary tumour RNAseq data from five public repositories, pooling samples to maximise statistical power and integrating data by correcting for technical variance. Data were split into train and validation sets. The train dataset underwent variable selection via a 10-fold cross-validation process that combined three algorithms in 100 models per fold. Genes found in at least 80% of models and five folds were considered robust to build a consensus multivariate model. A random forest model was constructed using selected genes from the train dataset and tested in the validation set. We also assessed the goodness of prediction by recalibrating a model using only the validation data. The biological context and relevance of signals was explored through enrichment and pathway analyses using QIAGEN Ingenuity Pathway Analysis and GeneMANIA.

**Results:**

We developed a pipeline that can detect robust signatures to build composite biomarkers. We tested the pipeline in PDAC, exploiting transcriptomics data from different sources, proposing a composite biomarker candidate comprised of fifteen genes consistently selected that showed very promising predictive capability. Biological contextualisation revealed links with cancer progression and metastasis, underscoring their potential relevance. All code is available in GitHub.

**Conclusion:**

This study establishes a robust framework for identifying composite biomarkers across various disease contexts. We demonstrate its potential by proposing a plausible composite biomarker candidate for PDAC metastasis. By reusing data from public repositories, we highlight the sustainability of our research and the wider applications of our pipeline. The preliminary findings shed light on a promising validation and application path.

**Supplementary Information:**

The online version contains supplementary material available at 10.1186/s12911-024-02578-0.

## Background

Advances in computational biomarker discovery have significant implications for biomedical research and are playing an important role for diagnosis and personalised treatment of patients [1, 2]. Machine learning (ML) based methods have been widely applied to biomedical research, especially cancer research [3]. However, ML-based methods face a data leakage and reproducibility crisis, with poor practices affecting numerous studies [4]. Inappropriate data handling and poorly designed workflows can lead to faulty and over-optimistic results. Moreover, variations in input parameters and sample variability from a target population can lead to vastly different predicted outcomes, leading to identification of inconsistent biomarker candidates [5]. Controlling these variabilities to extrapolate true biological signal, consequently establishing a consensus in the variable selection process is paramount for identifying robust biomarkers. This is particularly important when using biological data derived from omics experiments (e.g. transcriptomics, proteomics, or metabolomics) as a low variable number to sample ratio and high variance between experimental batches is common and can impact negatively potential results, normally requiring statistical corrections for data integration. Moreover, in clinical studies, is not uncommon to face a shortage of control subjects, which could result in class imbalance, which most off-the-shelf methods are not designed to handle. These challenges emphasise the need for a consistent analytical framework for biomarker discovery, to produce reliable and robust results.

By focusing on well-known health problems, our research aims to make a meaningful contribution to the methodology and understanding of a critical biomedical issue. Pancreatic ductal adenocarcinoma (PDAC) was selected to be explored in this study. PDAC is a highly aggressive cancer and a high potential for metastasis [6], which makes treatment challenging [7]. Previous work has focused on finding biomarkers to detect PDAC tumours at an early stage, because the majority of PDAC cases (80–85%) are diagnosed with advanced or metastatic disease, with only 15–20% eligible for potentially curative resection. [8]. The 5-year survival rate for PDAC patients with metastatic disease is only 5–10% [9].

Despite these challenges, many recent studies have been conducted on PDAC using ML, including risk prediction, early diagnosis, pancreatic fistula after pancreaticoduodenectomy, pathological tumour response to neoadjuvant chemotherapy, molecular subtyping, and microsatellite instability status [10].

There have been numerous attempts to achieve early detection using ML. For example, a study applied AI methods to clinical data to predict the risk of pancreatic cancer, showing promising results beneficial for future disease screening [11]. A three-dimensional convoluted neural network (3D-CNN) was used in PDAC diagnosis, predicting lymph node metastasis and the postoperative positive margin status based on preoperative CT scans [12]. ML models such as tree regression and logistic regression were implemented in clinical decision making in surgery of early-stage PDAC patients [13]. Gene expression biomarker signatures have been reported in other cancers such as colorectal cancer progression but have not been effective for PDAC [14]. Despite these advancements in disease surveillance, diagnosis, and treatment, PDAC progression remains challenging to manage and warrants further studies.

Early detection of PDAC is difficult due to asymptomatic or nonspecific symptoms in the early stages, resulting in patients being ineligible for surgical resection when clinical symptoms appear [15]. Diagnostic imaging is useful tool to diagnose PDAC early but may miss early-stage cancer before symptoms appear. Surveillance programs for high-risk patients are necessary, but screening asymptomatic individuals is challenging due to potential false positive results and costs [16]. The FDA-approved diagnostic biomarker for PDAC, serum carbohydrate antigen 19-9 (CA 19-9), has limited effectiveness for this purpose [17, 18]. Most diagnosed PDAC patients already have metastatic cancer and even those undergoing surgical resection may succumb to metastatic disease since they already have with clinically undetectable micro metastases at the time of surgery, presenting detectable tumours later [19, 20]. Finding reliable biomarkers to predict PDAC metastasis remains an unmet need and could help to identify patients in need of anti-metastatic therapies, thereby improving patient outcomes [21].

Here, we propose a machine-learning in-silico biomarker discovery pipeline that can be applied to multiple disease contexts. We showcase how it applies to integrating gene expression data to identify biomarker candidates for PDAC metastasis. Our robust workflow follows ML best practices, including feature selection, resampling techniques, and classification models, to generate consistent and reliable biomarker candidates for metastatic PDAC patient classification. We then contextualise the markers identified by examining their biological function. These biomarker candidates can be investigated further in laboratory and validated in clinical studies in the future.

## Methods

### Data preparation and inclusion criterion

In this study, we investigated PDAC datasets from all major public repositories, including The Cancer Genome Atlas (TCGA) [22], Gene Expression Omnibus (GEO) [23], the International Cancer Genome Consortium (ICGC) databases [24], and The Clinical Proteomic Tumour Analysis Consortium (CPTAC) [25]. We applied a filter-out selection criterion to the data based on the following parameters: samples collected from primary tumour tissues of unpaired PDAC patients only, datasets containing patient clinical data for lymph node and distant metastasis, age and sex, and data acquired from RNA sequencing platforms. All datasets were processed from data summarised at the gene level, with exception of CPTAC which only raw counts were available. The patient stratification was summarised in Figure S1 in Additional file 1.

The datasets comprised PDAC cases with and without metastasis. We stratified the samples into two groups based on their metastasis status to local lymph nodes using AJCC cancer staging, including pathologic tumour and TNM staging where T is the size of the tumour, N is local lymph node invasion and metastasis, and M is distant metastasis [26]. We use these definitions: the samples from stage IA to IIA PDAC patients with no regional lymph node metastasis (N-0) were categorised as the “non-metastasis group”. In contrast, the “metastasis group” included stage IIB to IV PDAC patients. Our analysis utilised five datasets, as summarised in Table 1. We used TCGA-PAAD, PACA-AU and PACA-CA [27–29] as our train dataset for the variable selection steps for train/test processes and CPTAC-PDAC and GSE79668 [30, 31] as the validation datasets (accessed 10/10/2021).

**Table 1:** Patient Stratification and Sample Sizes - This table presents the stratification of samples based on cancer stages. The samples are divided into two classes: metastasis (involving lymph nodes and/or liver) and non-metastasis. Stages IA to IIA are categorized as non-metastasis, while stages IIB to IV are classified as metastasis. The table displays the count and percentage representation of each class in train and validation datasets. The metastasis and non-metastasis groups are imbalanced

***Tumour stage***	***T***	***N***	***M***	***Class***	***Train data (%)***	***Validation data (%)***
*IA*	T1	N0	M0	Non-metastasis	107 (34.74)	43 (23.75)
*IB*	T2	N0	M0			
*IIA*	T3	N0	M0			
*IIB*	T1-3	N1	M0	Metastasis	201 (65.26)	138 (76.25)
*III*	T4	N0, N1	M0			
*IV*	Any	N0, N1	M1			
				Total no. of samples	308	181

### PDAC data pre-processing and integration

Data analyses using R version 4.2.2 [32] involved normalisation, gene filtering, identifying consistent expression patterns, dataset merging, and batch effect removal. The dataset showed vast differences based on technical variance (see Figures S2 and S3 in Additional file 1). Trimmed Mean of M-values (TMM) normalisation was applied using the edgeR package [33] to account for sequencing depth and composition differences between samples [34]. Genes with low expression levels (< 5% quantile & < 0.1 Absolute Fold Change) were filtered out. To address batch effects, which introduce unwanted variability between experiments, we used ARSyN (ASCA removal of systematic noise) mode 1 (batch correction only) from the MultiBaC package [35]. This method requires a lower number of features to converge. This reduction was undertaken by filtering out genes that did now show a consistent expression pattern across technical batches in the train dataset. Batch correction was undertaken on both train and validation datasets together to transform the datasets to allow biological signals to be identified, given the large technical variance.

### Biomarker candidate identification process

#### Analysis workflow

**Fig. 1:**
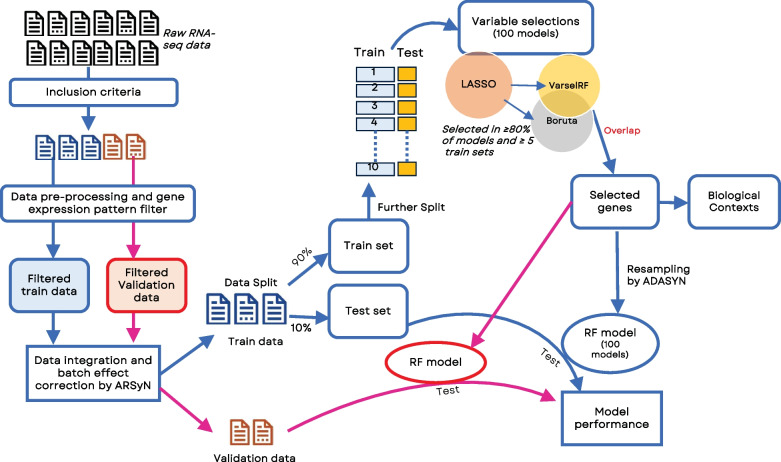
Analysis workflow, including data pre-processing, variable selection, re-sampling technique, and classification model

We designed an in-silico workflow for biomarker discovery to predict metastatic tumour stages as a binary outcome using ML (see Fig. 1). After data processing, data was split into train and validation data. The train data was further split into a 90% train set and a 10% test set. The train data underwent 10-fold cross-validation, using Least absolute shrinkage and selection operator (LASSO) logistic regression, glmnet package [36] for variable selection first, then selected variables from LASSO were further selected by Boruta [37], and the backwards selection algorithm within package varSelRF [38] on 100 models per fold. Genes found in at least 80% of models per fold and five folds in both Boruta and varSelRF were considered robust marker candidates.

We used random forest models [39] implemented via the ranger method in caret package [40], an ensemble of decision trees for improved classification accuracy, 5-fold cross-validation, and oversampled by ADASYN [41] to balance the proportion of classes in all modelling approaches. The train set was used to build 100 models (Fig. 1, blue line), while the test set was used to assess each model shown in Fig. 1. The details of criteria and parameters used in each of these methods, together with all the code for this pipeline can be consulted in ML_PDACBiomarker GitHub repository [42].

#### Model evaluation

The Random Forest models were evaluated using a comprehensive set of twelve metrics, specifically chosen for their effectiveness in analysing imbalanced data. These included Precision, Recall, and F1 score for both the metastasis (class 0) and non-metastasis (class 1) classes, which assessed the models’ performance in correctly identifying instances of each class. To provide a balanced view of the models’ overall performance, Macro-Averaged Precision, Recall, and F1 score were also calculated, giving equal weight to each class [43]. The model’s ability to distinguish between the two classes at various threshold settings was quantified using the Receiver Operating Characteristic - Area Under the Curve (ROC_AUC) [44]. The Precision-Recall - Area Under the Curve (PR_AUC) was used to measure the model’s precision-recall trade-off at different threshold settings, a metric particularly useful for imbalanced datasets [45]. Lastly, the Matthews Correlation Coefficient (MCC) provided a balanced measure of the model’s performance, considering true and false positives and negatives [46]. Further details of evaluation metrics and their calculations can be found in Additional file 2 part 1.

#### Hyperparameter tuning

RF models require tuning of hyperparameters such as *ntree* (number of trees in forest)*, mtry* (number of features considered for splitting at each node)*, splitrule (rule for node splitting),* and *min.node.size (minimum number of observations required for a node to split)*. Hyperparameter tuning was undertaken with the train function from the caret R package [40], selecting the method as ranger. We tuned the hyperparameters using a grid search approach with 10 different combinations of values, facilitated by the tuneLength = 10 parameter. Additionally, we employed a 5-fold cross-validation (CV) strategy to ensure the generalizability of the model’s performance across different subsets of the data. Further details of hyperparameters can be found in Additional file 2 part 2.

### Model performance comparison

#### Model performance comparison with XGBoost

We compared the performance of the RF model created with the genes selected against an equivalent one using XGBoost (XGB), both of which are popular decision tree algorithms. We built XGB models using the same procedures as the RF model. We employed the train function from the caret R package [40], selecting the method as ‘xgbTree’ [47]. Hyperparameter tuning was performed with a range of tune grids, following hyperparameters: nrounds (the number of boosting rounds), max_depth (the maximum depth of a tree), eta (the learning rate), gamma (the minimum loss reduction required to make a split), colsample_bytree (the fraction of columns to be randomly sampled for each tree), min_child_weight (the minimum sum of instance weight needed in a child), and subsample(the fraction of observations to be randomly sampled for each tree).We applied the XGB model to the training data, performed validation (a model trained by train data and tested by validation data), and model in validation data. The results are presented in Tables S11-S13 (Additional file 2 part 3).

#### Model performance comparison between 15 selected genes and 15 random genes

We implemented the same analysis approach for validation using 15 randomly selected genes from the data matrix used for variable selection. A Random Forest (RF) model was trained on the train data and subsequently tested using validation data, as depicted in Figure 7S (Additional file 2 part 4). This process was repeated 100 times to ensure robustness. The performance of the models were recorded for the same 12 metrics outlined above, and the mean and 95% confidence interval were calculated to provide a comprehensive view of their performance shown in Figure 8S (Additional file 2, part 4).

### In-silico validation and biological contextualisation

#### In-silico validation

To validate the predictive potential of our composite biomarker candidate, we created random forest models using selected genes from the train dataset and test their predictive capacity on the validation dataset (see Fig. 1 pink line). The optimal hyperparameter configuration, as determined by the highest accuracy on the training set, included *ntree* = 500, *mtry* = 3, *splitrule* = extratrees, *min.node.size* = 1. We also assessed the goodness of fit on the validation data using a recalibration approach. Splitting the validation data into a 90% train set and a 10% test set; we created 100 new RF models with the candidate composite biomarker on the train validation data evaluating model performance on the test data, the model performances were averaged from these 100 models. This was done to ensure that the predictive capability of the selected biomarker candidates was stable and not dependent on a particular random seed or data split.

#### Biological contextualisation

The selected genes were explored in their biological context using bioinformatics tools: QIAGEN IPA [48], GeneMANIA [49], and The Human Protein Atlas or HPA [50]. We used GeneMANIA in modes for gene function prediction and network analysis of the candidate biomarker list. Composite biomarker candidate -fifteen genes (set1) were inputted into the GeneMANIA plugin version 3.5.2 of Cytoscape version 3.1.1 software [51]. The software allowed setting the advanced options, *H. sapiens* (Human), and query gene-based weight was set. The attributes e.g., drug interaction and miRNA targets were unchecked as we focused on gene–gene network. The top 20 relevant genes based on weight were added to construct a gene network. Biological processes were assessed by gene ontology enrichment analysis with statistics corrected for false discovery rate. QIAGEN Ingenuity Pathway Analysis (QIAGEN IPA) is a web-based software application that facilitates the analysis, integration, and understanding of data from various omics experiments. We utilised QIAGEN IPA for pathway and functional analysis. To further connect our analysis results with additional biological findings, we analysed the secretome of low and high metastatic murine pancreatic cancer cell lines using Stable Isotope Labelling by Amino acids in Cell culture (SILAC) as explained in Luckett et al., 2024 and identified 34 proteins upregulated in the highly metastatic PDAC cells compared to the low metastatic PDAC cells [52].

We tested the differential expression of the mouse secretome using the Limma package [53], with an adjusted *P* < 0.05 and |Fold Change|> 1.0. We identified 37 differentially secreted proteins (set2) and 34 exclusively secreted proteins (set3) in the high metastatic group. Using QIAGEN IPA, we undertook core analysis using variables from sets 1–3 as listed in Additional file 3, with the user data set as a reference. The canonical pathways and network analysis are provided in Tables 5S-8S in Additional file 4.

We also tested protein-level data for the genes selected with our approach. Each gene was individually searched on the human protein atlas [50, 54]. General HPA information was summarised (see Table 1S in Additional file 1), including characteristics of a protein class, pancreatic cell type enrichment, and antibody staining in PDAC samples.

## Results

### Selected genes and model performance

Using the workflow described in a methodology, we selected fifteen genes that met the filtering criteria and were consistently selected by the three variable selection methods shown in Table 2 to predict metastatic tumour stage. Gene distribution by class is shown in Figure S4 of Additional file 1. We used pre-selection by LASSO to reduce the computational complexity and capture any linear relationships of genes with the patient class. Generally, LASSO can be computed very quickly and produces a larger pool of selected variables (more than 40 genes after filtering criteria). We then applied Boruta and varSelRF in LASSO-selected genes, capturing non-linear relationships among genes. Boruta and varselRF are wrapper methods within RF models for variable selection in high-dimensional datasets. These computations are more intensive and benefit from the reduced variable list, which also benefits the sustainability of the pipeline (with simpler calculations contributing to environmentally mindful science and lower energy consumption). Notably, Boruta selects these 15 genes in 10 out of 10 train splits. The algorithm finds all relevant attributes, not finding a minimal-optimal set of genes as LASSO and backwards selection. It is designed for dealing with complex data, e.g., cancer gene expression data, which is needed to find all cancer-related genes [37]. While further improvements and reductions of the candidate composite marker may be possible, we took forward the most robust but conservative set.

**Table 2:** Top variable selection frequencies with different algorithms, numbers indicate number of folds out of 10 in which the gene was selected in at least 80% of models

***Gene***	***LASSO***	***BORUTA***	***VARSELRF***
ABCC9	10	10	10
AP1M2	10	10	10
CAPN5	10	10	10
ITK	10	10	10
MPDZ	10	10	10
RCSD1	10	10	6
TMPRSS4	10	10	10
ABCA8	9	10	9
ELMO3	9	10	5
C1R	8	10	10
CELF2	8	10	10
IL7R	8	10	10
TTYH2	8	10	9
CD37	7	10	9
ZFP82	7	10	8

#### Train data model performance

One hundred random forest models were created with these genes and assessed in different data splits in model training and testing in individual train data. The model performance in the train data was, as expected, very good in all evaluation metrics shown in Table 3.

**Table 3:** performances on train data (on teh train/test splits) showing average values and 95% confidence interval on the validation data (100 models)

**Metric**	**Mean**	**95% CI**
PC0	0.925	[0.872;0.978]
RC0	0.889	[0.826;0.952]
F10	0.904	[0.845;0.963]
PC1	0.804	[0.725;0.883]
RC1	0.847	[0.775;0.919]
F11	0.816	[0.739;0.893]
MPC	0.864	[0.796;0.932]
MRC	0.868	[0.800;0.936]
MF1	0.860	[0.791;0.929]
ROC_AUC	0.945	[0.899;0.991]
PR_AUC	0.894	[0.832;0.956]
MCC	0.731	[0.642;0.820]

*PC* Precision, *RC* Recall, *M* Macro average, *PR_AUC* Area under the curve of pr curve, *ROC_AUC* Area under the curve of roc curve, *MCC* Matthews correlation coefficient, *0* Metastasis, *1* Non-metastasis, *CI* Confidence interval

#### Validation data model performance

The model build on the train dataset was assessed on its prediction power on the validation data. We also recalibrated the model on the validation data on train/test splits. Performances for both approaches in Table 4. The results exemplify the predictive capacity of the candidate biomarker list that could set basis for a promising composite biomarker.

**Table 4:** RF model performances of a model trained by train data and tested by validation data (second column) and, RF model performances showing average values and 95%CI on the validation data (100 models, third and fourth columns)

**Metrics**	***Cross-dataset validation performances***	***Re-calibration validation performances***	
		**Mean**	**[95%CI]**
PC0	0.946	0.928	[0.876;0.980]
RC0	0.884	0.971	[0.937;1.000]
F10	0.914	0.947	[0.902;0.992]
PC1	0.692	0.911	[0.854;0.968]
RC1	0.837	0.740	[0.652;0.828]
F11	0.758	0.793	[0.712;0.874]
MPC	0.819	0.919	[0.864;0.974]
MRC	0.861	0.855	[0.785;0.925]
MF1	0.836	0.870	[0.803;0.937]
ROC_AUC	0.839	0.941	[0.894;0.988]
PR_AUC	0.938	0.897	[0.836;0.958]
MCC	0.678	0.765	[0.680;0.850]

#### Model performance comparison

We performed two comparison including model type (RF and XGB) and selected genes (15 final genes and 15 random genes). We applied the XGB model to the training data, performed cross-dataset validation (a model trained by train data and tested by validation data), and re-calibration validation data. The results are presented in Tables S11-S13 (Additional file 2 part 3). Briefly, RF model outperformed marginally XGB in almost all evaluation metrics and in the three modelling approaches, but results were reasonably comparable, which further highlights the biological signal potential of the biomarker candidate list.

The RF model performs drastically better when trained on the 15 selected genes compared to the 15 random genes as shown in in Table 14S (Additional file 2 part 4). Random genes, poorly performed in almost all metrics (Figure 8S in Additional file 2) – note accuracy could appear high in some runs but it is coupled with a majority class “guess” and linked to a low MCC. These results suggest that the 15-gene signature reported here, as consistently outperforms the model trained on the random genes across all metrics, likely contains biologically relevant information.

### Biological contextualisation

The results of gene ontology enrichment analysis in GeneMANIA are summarised in Table 3S in Additional file 4. The gene network was created as shown in Figure 5S in Additional file 4. The details of nodes and edges of gene network can be found in Tables 3S and 4S in Additional file 4. The gene network revealed highly enriched immune response-associated pathways, as shown in Table 2S in Additional file 4.

**Fig. 2:**
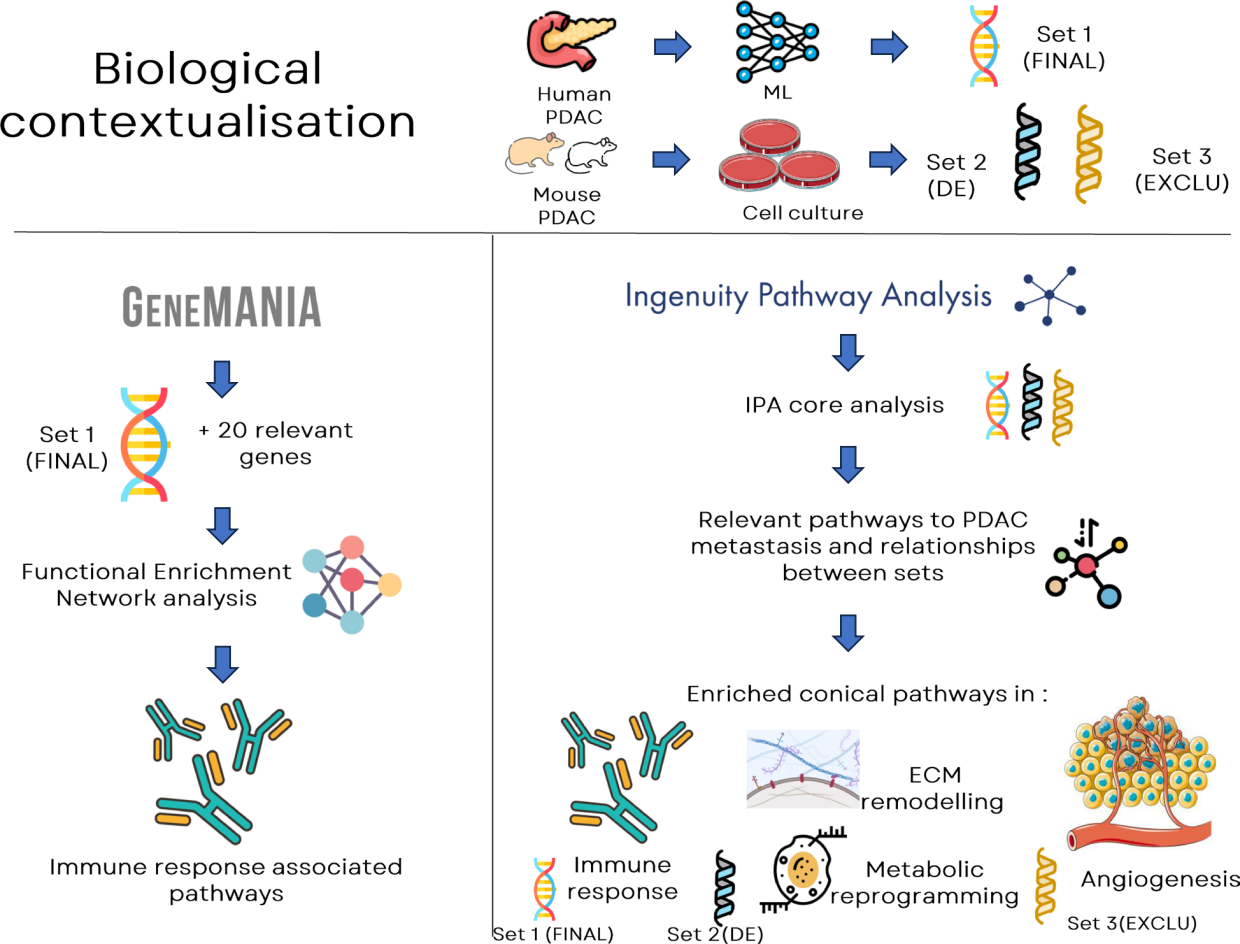
Biological contextualisation using GeneMANIA and QIAGEN IPA: set 1 showed immune-related pathways enrichment, while sets 2 highlighted EMC remodelling & metabolic reprogramming and set 3 enriched angiogenesis

Further investigations were done by running core analyses of gene/protein sets 1–3 in QIAGEN IPA. Canonical pathways of set 1 showed highly significant immune responses, consistent with GeneMANIA results. While the results of set 2 were related to cancer metastasis, including extracellular matrix (ECM) remodelling and metabolic reprogramming, set 3 showed additional biological insights into angiogenesis, as shown in Fig. 2. These are processes involved in metastatic development.

**Fig. 3:**
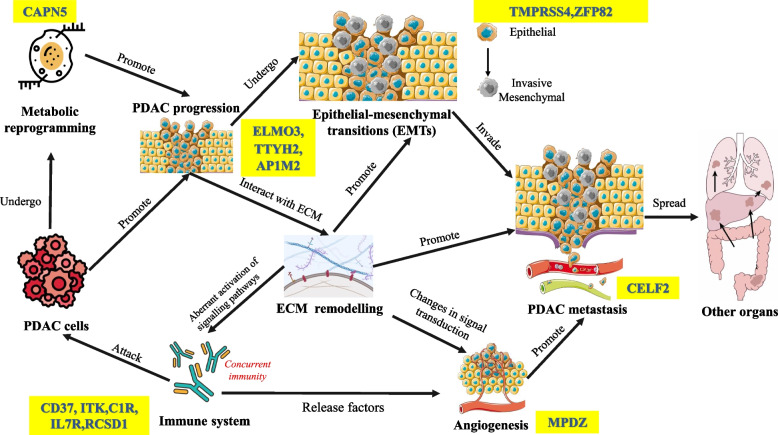
The complex and multifaceted interplay between immune response, extracellular matrix remodelling, metabolic reprogramming, and angiogenesis in cancer metastasis including PDAC (Biomarker candidates involved in biological processes are highlighted in yellow)

To focus on our findings from ML, we conducted a detailed literature review on the 15 genes, which is graphically represented in Fig. 3. The schematic shows the complex and multifaceted interplay between immune response, ECM remodelling, metabolic reprogramming, and angiogenesis in PDAC metastasis. Many genes of our findings play import roles in PDAC progression and metastasis.

Further exploration at the protein level was undertaken using the Human Protein Atlas database. The aim was to explore further the potential for this composite biomarker candidate and the feasibility of experimental validation. Several genes of interest, reported as disease-related and associated with drug targets, were identified. These genes exhibited enrichment in both immune and non-immune cells. Antibody staining in pancreatic ductal adenocarcinoma (PDAC) samples, validated by TCGA data, demonstrated varying intensities for certain genes, ranging from low to high and weak to strong. While some genes were extensively investigated at protein level, others remain unexplored. The results from the HPA were summarised in Table S1 in Additional file 1.

### A generalised workflow for in-silico biomarker discovery

We have presented an example workflow applied to PDAC metastasis for identifying in-silico biomarkers following best practices in ML. In developing this workflow, we had to appraise multiple steps, overcome challenges, and compare methods. We have collated our findings and recommendations in the general workflow shown in Fig. 3. This workflow can be applied to any in-silico biomarker candidate discovery project, especially those employing omics technologies. Some of the main challenges to overcome in these processes are the typically small sample sizes, the vast technical variations typically observed in biological/clinical experiments and how to integrate data without invalidating the independence of train and test data splits. Based on our experience we are proposing these recommendations. Data sources should be kept consistent and undergo a selection criterion defined by the research question and the data types available for analysis (aiming to select data from the same technological platforms for each layer of biological complexity – for example, gene expression analysis should include data from either microarray or RNAseq technologies but avoid integration from both sources due to vast technical variations). Data pre-processing is key to data integration and further downstream analysis. Data should be processed via the same normalisation pipeline to minimise technical variance but aiming to keep independence of the various data sets; normalisation methods depend on the technology of choice; some commonly used are Variance Stabilisation Normalisation (VSN) in proteomics [55], Probabilistic Quotient Normalization (PQN) in NMR metabolomics [56] or TMM for RNAseq [34]. Despite being commonly used, it is good to appraise different methods for each project and select the most appropriate one based on unbiased metrics (for example, by appraising empirical housekeeping variables, the variance of which should be minimised by the best normalisation method). All variables too close to the limit of detection should be discarded for biomarker discovery.

Data may also be transformed (for example, log-transformed) and scaled to control for the effects of variable magnitude in model parametrisation, which is relevant in omics analyses [57]. This process is essential in some multivariate modelling as parameter sizes affect selection (while in most cases, we aim to detect the largest differences between groups independently of the specific magnitude of the variable). Different aspects need to be considered, including assessing technical effects and (if appropriate) batch correction. Technical and biological effects can be appraised with methods such as Principal Variance Component Analysis (PVCA) [58] or variancePartition [59]. These methods can inform on the main sources of variance the data accounts for, which will help appraise different modifications and select which covariates are most appropriate to include in modelling. Batch corrections should be undertaken with methods designed to remove known batch variance only. Some examples are Combat [60], ARSyN – mode 1(batch correction only) [35] or RUVseq [61]. It is important to avoid methods that remove all variance unrelated to the interest grouping, such as SVA [62] or ARSyN – mode 4 (batch correction and noise reduction) [35]. These methods remove all variance not linked to the group of study, which can be particularly dangerous as this will invalidate the independence of train/test data if both train/test datasets undergo this step and may remove important biological variance unknown with experimental groups but relevant to the question at hand. Finally, missing values should be appraised if they exist with particular attention on whether they are missing completely at random, missing at random and missing not at random. Variables that pass certain control threshold for missingness could go through imputation with methods such as missForest [63] or multiple imputation by chained equations [64]. It is advisable to evaluate different methods in the process.

One of the most key steps for ML-based biomarker discovery methods is to control data leakage [65]. It is then important to split all data available into a train set that may be used for further analysis and a validation set reserved to assess the final models (and not used for any selection). The train set can undergo variable selection. Variable selection should be undertaken applying methods such as bootstrapping or N-fold cross-validation stages to limit the influence of particular samples’ variance (for example, patient heterogeneity) in identifying consensus variables to detect the outcome of interest. This step involves creating different data slices from the train data (train/test) to assess variable selection results. The most relevant discriminatory variables will be consistently selected in all data slices, producing a candidate consensus signature. Different variable selection methods are advised to ensure that only robust and consistent signatures are taken forward. Some examples of variable selection methods are shown in this project; for example, implicit methods such as LASSO logistic regression penalises highly correlated variables; explicit methods such as varselRF, which is good at identifying non-linear relationships; and Boruta which finds relevant features by comparing original attributes and its permuted copies (shadows) [37]. 

**Fig. 4:**
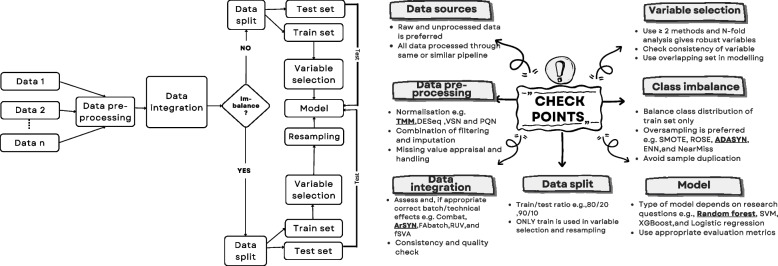
Proposed in-silico biomarker discovery workflow and checkpoints to consider (implemented methods in GitHub pipeline are bolded and underlined)

It is also common to face class imbalance issues in clinical data. Different techniques can be used to deal with this issue; the Synthetic Minority Over-sampling Technique (SMOTE) [66] is one such method that balances the dataset by creating synthetic instances of the minority class. Another technique, Resampling Over-sampled Examples (ROSE) [67], also focuses on oversampling but uses a distinct algorithm to generate synthetic samples. The Adaptive Synthetic Sampling (ADASYN) [68] method addresses class imbalance by prioritising the generation of synthetic instances in areas where the imbalance is most severe. These strategies are instrumental in boosting the performance of machine learning models when dealing with imbalanced datasets in clinical settings. It is important to use these techniques only on the train data and ensure variables are not duplicated by using these techniques. Finally, the model type selection should be suitable to the research question with different choices for classification or regression. Random forest is particularly useful as it is versatile (both classification and regression models possible). It is robust with limited overfitting thanks to the combination of multiple decision trees in different data folds, and it is interpretable, providing insights into which variables are most important for the model. Random forest has been used in several studies to solve biological and medical problems, including biomarker identification in cancer, particularly in analysing cancer gene expression data [69]. In Fig. 4 we summarise the key aspects to consider when undertaken in-silico biomarker discovery.

## Discussion

Our study proposes a robust pipeline for identifying biomarker candidates using ML and legacy data. The workflow was applied to PDAC data to find metastatic biomarker candidates. We collected and curated data from major repositories. Data were integrated and run through our workflow, giving fifteen genes consistently found in different data slices and via three variable selection algorithms, performing very well in classification models.

We have solved issues in data analysis using ML and proposed an in-silico biomarker discovery workflow that can be applied to other contexts. Our analysis workflow was designed with the best practices suggested by Kapoor and Narayananm 2023 in mind. Our approach emphasises the importance of addressing leakage issues and ensuring the reproducibility of the machine learning-based model [4]. It also addresses challenges that are particularly difficult to overcome when exploiting omics data, which are inherently variable due to technical differences. Such leakage can cause the model to overestimate its accuracy and irreproducible results. While this study has limitations regarding the step of data integration, as much as was possible, we implemented a clean separation of training and validation datasets in our study. Importantly, variable selection and resampling techniques were applied in only the train set. Our machine learning workflow faced challenges in data integration due to the need to apply batch effect corrections to all data. While the most purist way of addressing these technical disparities while mitigating data leakage would have been to correct these effects separately in the train and validation sets, this was insufficient to bring all data on the same geometrical space to aid the integration and testing of predictive power. Cross-study of medical data uses train data to build models and external data to test them, and often leads to poor prediction performance due to a lack of comparability (39). We acknowledge this is a limitation of our study but something we cannot overcome if we aim to retain data and power. We mitigated this data integration and batch correction step by using methods that removed variance about technical platform/experiment batch only and ensured all batches represented both groups of interest (metastasis and non-metastasis). Our model demonstrated robust performance, with an MCC of around 0.7, indicating its strong predictive performance and potential as a reliable composite biomarker candidate for patient classification in metastasis.

In this study, we collected data from major repositories to reuse and improve power for our research question. Using these repositories has several benefits: it promotes research sustainability by reusing valuable data, confirms previous findings when there is agreement, increases patient profile diversity, makes biomarker results more credible, and enhances the statistical power of the ML model through data integration. We acknowledge a major limitation of studying pancreatic primary tumour samples is the high heterogeneity of tumour cellularity (which, unfortunately, is not consistently reported across repositories and a limitation to accept as it currently stands for legacy data). We created a workflow tailored for end-users. Consequently, we provide comprehensive data and R code on GitHub, adhering to the FAIR Guiding Principles [70], allowing free use, modification, and sharing. With support from the scientific community, we ensure the accessibility and robustness of the developed workflow. Following a thorough review of ML-based data analysis, we designed an in-silico biomarker discovery workflow with checkpoints for beginners. We developed step-by-step guide to help with user accessibility and help them navigate common ML pitfalls and presented them as part of our results. This versatile workflow guides handling various data types, particularly omics data, and is not limited to PDAC metastasis analysis. It can be applied to diverse contexts, and this team has applied it to other problems such as identification of biomarker candidates of pancreatic cancer-related diabetes using proteomics data [71], biomarker discovery in adverse pregnancy outcomes [72, 73] or gene signature prediction in human breast cancer subtypes. The workflow presented here integrates algorithms for data integration, variable selection, resampling techniques, and modelling to enhance its overall robustness.

We assessed the model performance using a total of 12 metrics, including class-specific metrics, macro-average, AUC, and MCC. Our chosen metrics, such as precision, recall, F1, PR_AUC, and MCC, are particularly effective for evaluating models trained on imbalanced datasets [46, 74]. Interestingly, a biomarker study in lung cancer [75] and stress detection [76] employed similar metrics to ours, such as accuracy, precision, recall, F1 score, and PR AUC for model evaluation. However, they did not incorporate the MCC metric into their evaluation process. This highlights the thoroughness of our approach in considering a wide range of metrics for model evaluation.

One of the key components of our workflow is the variable selection process. In a novel use of selection techniques, we propose a new paradigm for composite biomarker selection, bringing forward only consistently and robustly selected signatures across different data folds and variable selection methods. This approach ensured the selection of the most relevant features for our biological question, and it aligns with current best practice in the biomarker discovery field [5]. We overcame the class imbalance problem using, ADASYN, a resampling technique that improved the learning of the random forest model, balanced sample proportion, reduced the sample distribution bias (25). The algorithm created newly synthesised elements without data duplication. Resampling techniques in imbalanced data typically consist of oversampling and undersampling. Oversampling is preferred in the context of medical data as it can produce a more diverse set of examples from the less represented class, thereby improving the model’s capacity to generalise [77]. The techniques used to address the imbalanced class problem directly contributed to the success of the RF model in classifying patients.

In this case study, we showed how our workflow could deal with a complex biological task. PDAC is a very aggressive cancer type with high metastatic potential and fast progression, therefore capturing the change of biological phenomena from non-metastasis and metastasis is challenging. Many biomarker studies using ML in data analysis mainly classify normal and cancer as biologically distinct due to the numerous changes that occur during tumour development [78]. Detecting metastasis in a fast progression disease as PDAC (T1 to T4 stage in just 1 year [79]), is challenging. We used primary tumour gene expression data, stratifying patients using cancer pathological staging and N (regional lymph node involvement) as we tried to capture metastatic events; stage IIA and IIB are closely staged but are biologically distinct. PDAC stage IIA has no regional lymph node invasion, while stage IIB has. Therefore, we used this change as our criterion to classify the patient samples, which differs from other previous works (22,23) that use cancer and normal samples instead. Human PDAC tumours are highly heterogenous and contain a host of cancer cell clones with varying metastatic fitness. Developing a technique sensitive enough to detect highly metastatic clones within the multiclonal population is challenging. It is expected to see groups of patients with very similar biological factors since they both have PDAC but with changes in biological processes caused by slight differential expression of some genes [80]. Thus, the observed small effect sizes in each variable are not of concern given the predictive power of the multivariate signature.

We further assessed the feasibility of the 15-gene composite biomarker candidate by biologically contextualising their role in PDAC progression. The data from the 15-gene composite marker (Set1) and secretome studies shed light on the underlying biological processes. Set1 linked to genes involved in immune responses, while the secretome data from the mouse experiment revealed proteins involved in extracellular matrix remodelling, metabolic reprogramming (set 2), and angiogenesis (set3).

The relationship between cancers, such as PDAC and other biological processes is complex, as presented in Fig. 3. Cancer cells undergo metabolic reprogramming to alter their metabolism, thereby gaining more energy for rapid proliferation. For instance, CAPN5 plays a significant role in glycolysis in pancreatic cancer [81] and endometrial cancer [82]. Cancer cells frequently exhibit an increased rate of glycolysis thus favouring the production of lactate, even in the presence of oxygen, in a process known as the Warburg effect [83]. Moreover, loss of ELMO3 is implicated in lymphatic metastasis in colorectal cancer [84], and in metastatic capacity in PDAC [85]. Additionally, TTYH2 and AP1M2 are implicated in cancer progression through regulation of the JNK/ERK Signalling Pathway in Hepatocellular Carcinoma. Furthermore, AP1M2 is linked to immunosuppression in the TME in various cancers [86, 87]. TTYH2 has also been described to promote cancer cell colony formation, thereby supporting tumour growth and metastasis in colon adenocarcinoma [88].

TMPRSS4 and ZFP82, have been shown to induce Epithelial-to-Mesenchymal transition (EMT) in pancreatic cancer [89, 90]. EMT is a biological process that plays a crucial role in cancer progression and metastasis, transforming otherwise immotile epithelial cells into migratory invasive mesenchymal cells. Several of our identified genes are related to the immune response in various cancer types, including PDAC. For example, both CD37 and RCSD1 are linked to recruitment of anti-tumour immune cells and improved prognoses [91, 92]. ITK is implicated in immune checkpoint process [93], C1R is an immunosuppressive complement effectors protein [94] and IL7R regulates in lymphocyte development [95]. In an in vitro study conducted by Tetzlaff et al., it was revealed that the protein MPDZ plays a role in promoting angiogenesis, a well-established process in metastasis, which is facilitated through the amplification of Notch signalling [96]. Finally, CELF2 promotes pancreatic cancer tumorigenesis and metastasis via Endoplasmic-reticulum-associated protein degradation (ERAD) [97]. While these observations are promising, we acknowledge that they require further validation to test their clinical translation potential. In PDAC, the early dissemination of primary tumour and clonal diversity of EMT at the metastatic sites need to be considered [6] as well as the links between resident immune and stromal cells and their role on the formation of a hospitable pre-metastatic niche (PMN) [98]. The tumour cellularity of samples also needs to be explored more. Single-cell RNA sequencing (scRNA-seq) analysis was reported to be a tool for studying PDAC heterogeneity and understanding in-depth molecular profiles [99]. Further studies exploiting scRNA-seq to understand metastasis mechanisms may aid the refinement of candidate markers and increase our understanding of the disease.

## Conclusions

Our study offers a machine-learning approach that identifies a robust and consistent composite biomarker candidate using PDAC metastasis as a case study. To ensure the reproducibility of our results, we applied various variable selection methods and resampling techniques to overcome the potential problems of machine learning in omics data. The general workflow and checkpoints in biomarker discovery using multiple data were designed to be a guideline for beginners. Our approach identified fifteen genes consistently present in all five datasets from different countries and performed well in classification models on both train and validation data. These genes have all been shown to potentially play a role in PDAC metastasis. Future studies are required to validate our composite biomarker candidate experimentally, with perhaps potential to refine it further. While our study has some limitations concerning joint data integration and limited sample size for validation, we believe we provide a robust candidate set to pursue further experiments and clinical work. We offer a thorough example of applying best practices in ML-based approaches for biomarker discovery in systems biology, and we tackle a challenging data integration problem. Although these findings need validation experimentally and in clinical studies, our study demonstrates how to identify effective biomarker candidates for the early detection and treatment of metastatic PDAC patients, accelerating translation potential.

### Supplementary Information

Additional file 1. Supplementary tables and figures.

Additional file 2. Model evaluation and comparisons.

Additional file 3. Gene and protein lists used in biological contextualisation.

Additional file 4. Results from GeneMANIA and QIAGEN IPA.

## Data Availability

The data has been reused from public repositories as reported in the methodology section. All the code is provided in GitHub Repository repository https://github.com/Victormah/ML_PDACBiomarker [42]. Additional files are provided as part of this submission, listed below references.

## Abbreviations

ADASYN: Adaptive Synthetic Sampling Approach
AI: Artificial Intelligence
AJCC: American Joint Committee on Cancer
ARSyN: Analysis of Variance Scale Components Removal of Systematic Noise
ASCA: Analysis of Variance-based Statistical Correction Algorithm
CA 19-9: Carbohydrate Antigen 19-9
CPTAC: The Clinical Proteomic Tumour Analysis Consortium
CT: Computed Tomography
EMC: Extracelullar matrix
EMT: Epithelial-to-Mesenchymal Transition
ERAD: Endoplasmic-reticulum-associated protein degradation (ERAD)
FAIR: Findability, Accessibility, Interoperability and Reusability
FDA: Food and Drug Administration
GEO: Gene Expression Omnibus
HPA: The Human Protein Atlas
ICGC: International Cancer Genome Consortium
KNN: K-Nearest Neighbours
LASSO: Least Absolute Shrinkage and Selection Operator
LIMMA: Linear Models for Microarray Data Analysis
MCC: Matthews Correlation Coefficient
ML: Machine Learning
PMN: Pre-metastatic Niche
PDAC: Pancreatic Ductal Adenocarcinoma
PVCA: Principal Variance Component Analysis
PR_AUC: Precision-Recall Area Under the Curve
PQN: Probabilistic Quotient Normalisation
RF: Random Forest
ROC_AUC: Receiver Operating Characteristic Area Under the Curve
RNA: Ribonucleic Acid
RNAseq: RNA sequencing
RUVseq: Remove Unwanted Variation in RNA-Seq data
ROSE: Resampling Over-sampled Examples
SVA: Surrogate Variable Analysis
SILAC: Stable Isotope Labelling by Amino acids in Cell culture
SMOTE: Synthetic Minority Over-sampling Technique
scRNA-seq: Single-cell RNA sequencing
TCGA: The Cancer Genome Atlas
TMM: Trimmed Mean of M-values
VSN: Variance Stabilisation Normalisation
XGB: XGBoost or Extreme Gradient Boosting
QIAGEN IPA: QIAGEN Ingenuity Pathway Analysis
