# Supplementary material for: Robust and consistent biomarker candidates identification by a machine learning approach applied to pancreatic ductal adenocarcinoma metastasis
Source: BMC Med Inform Decis Mak. 2024 Jun 20;24(Suppl 4):175. doi: 10.1186/s12911-024-02578-0 (PMC11191155; doi:10.1186/s12911-024-02578-0)
Supplement: Supplementary file 1 — Additional file 1. Supplementary tables and figures. [file 12911_2024_2578_MOESM1_ESM.docx]

**Supplementary data 1**


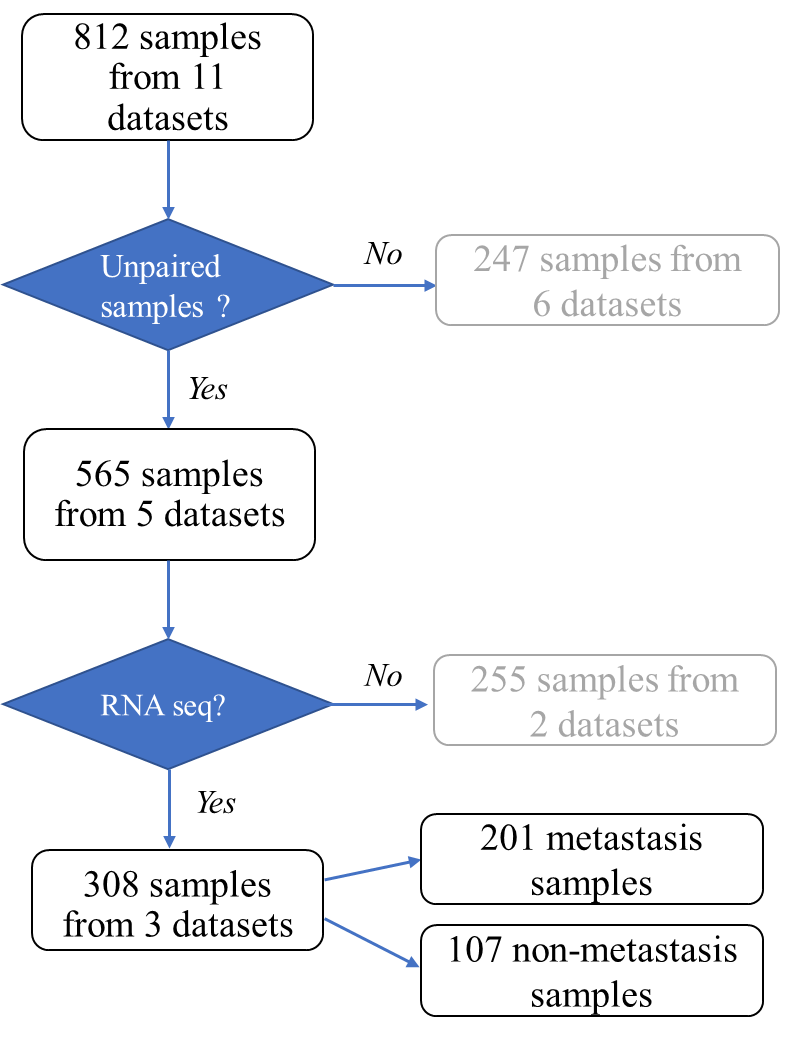

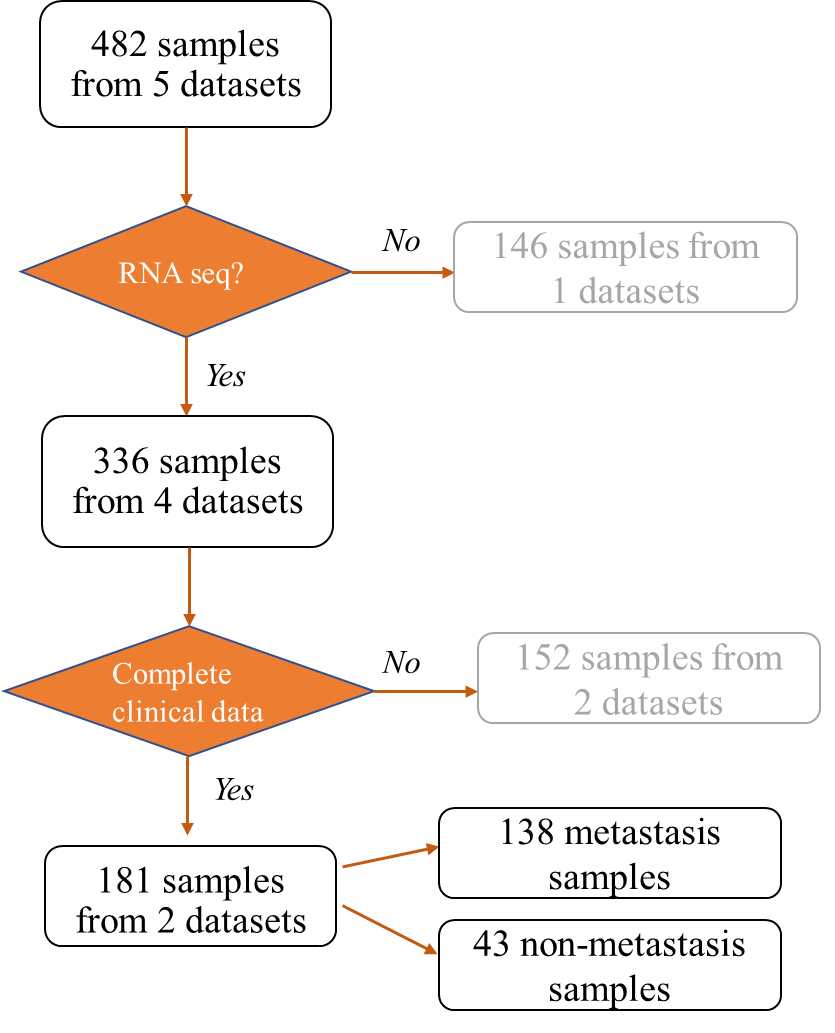


**A B**

**Figure S1**-Dataset selection flowchart, train data (A): 308 samples from 3 datasets and validation data(B) : 181 samples from 2 datasets were included after applying filter-out criteria.


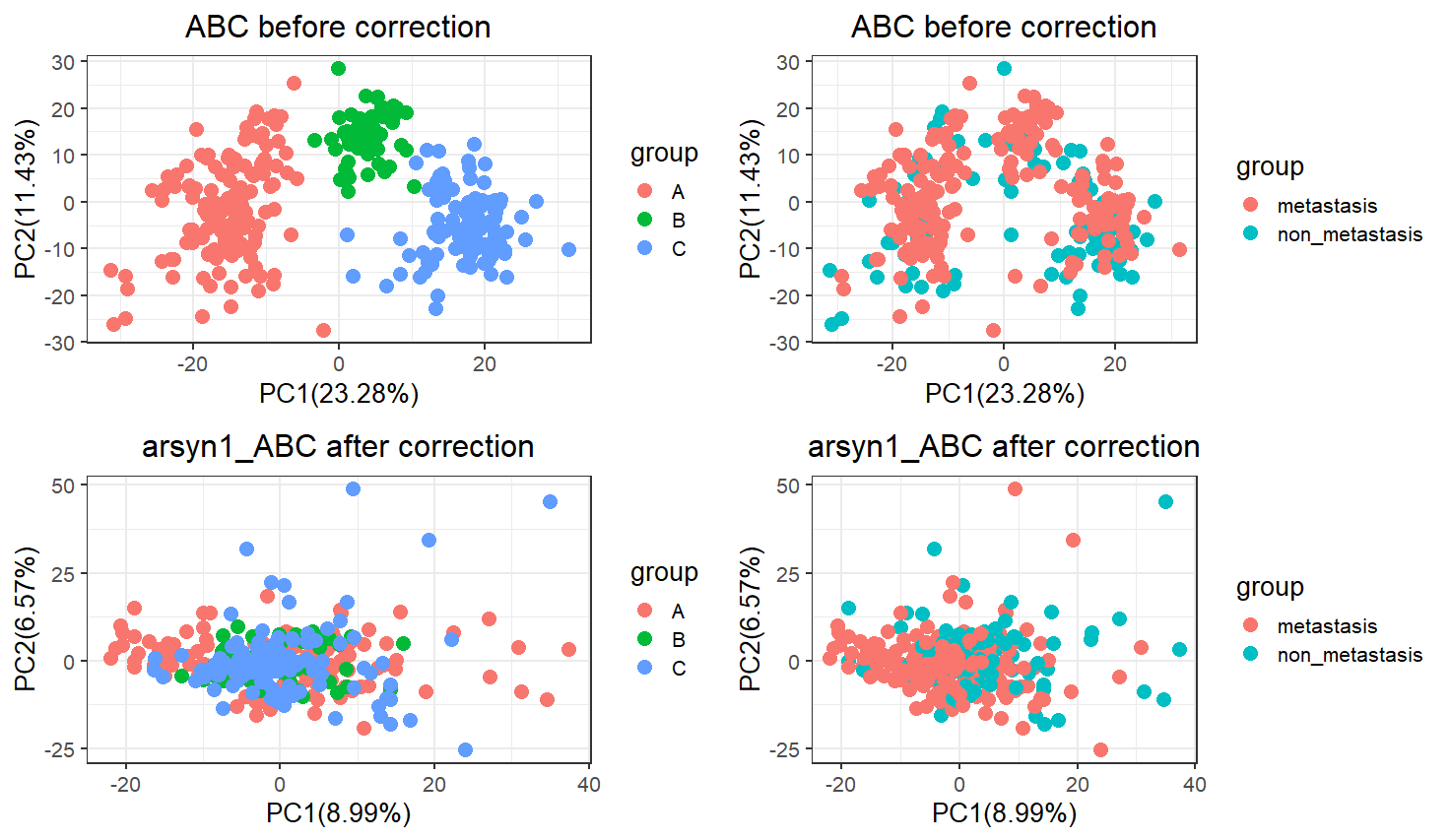


**Figure S2** : Train data (ABC) before (upper) and after ARSyN correction (lower) by data source : A =TCGA-PAAD,B= PACA-AU and C= PACA-CA ,and class of patients (metastasis and non-metastasis)


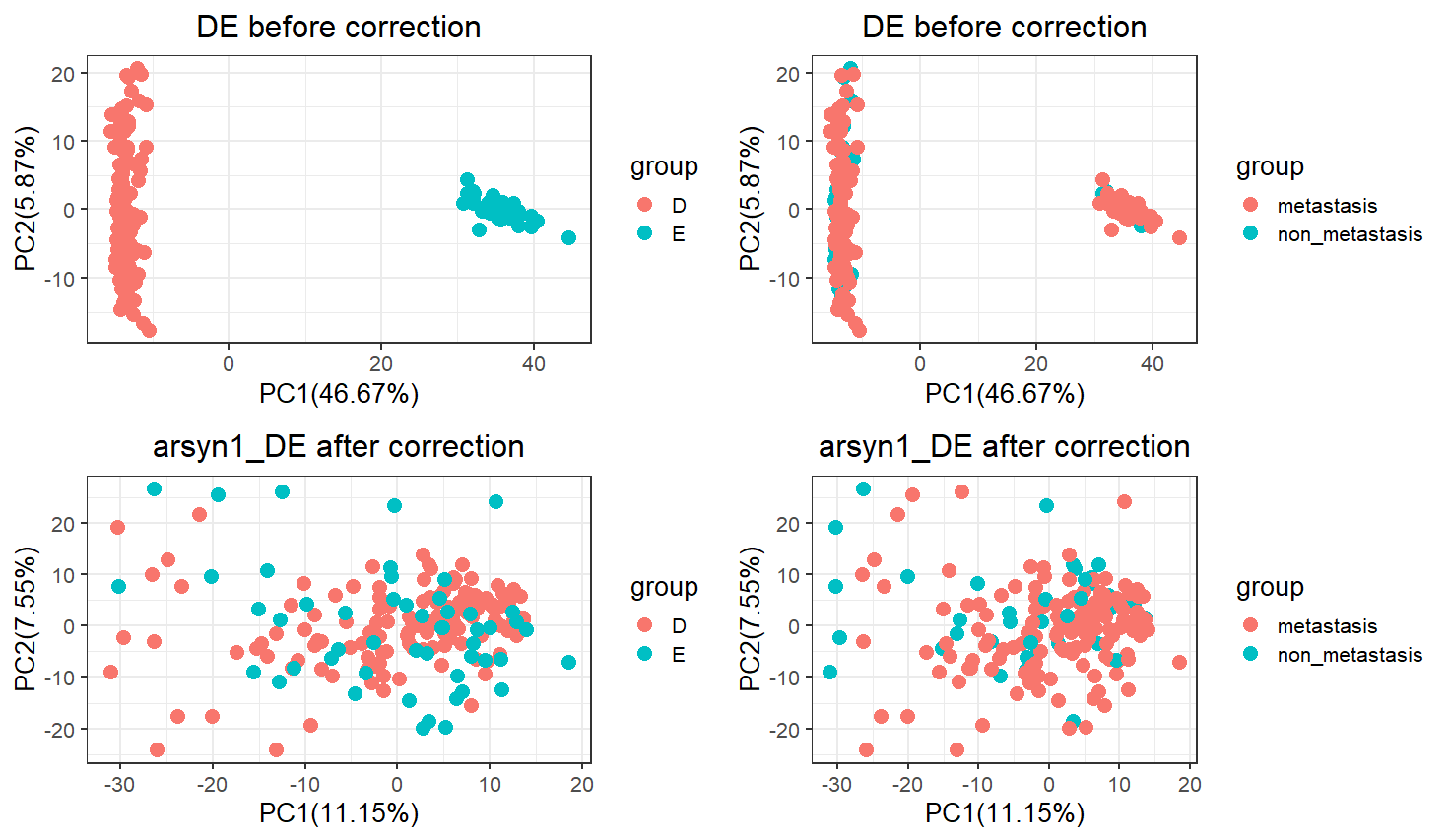


**Figure S3** : Validation data (DE) before (upper) and after ARSyN correction (lower) by data source : D= CPTAC-PDAC and E= GSE79668,and class of patients (metastasis and non-metastasis)


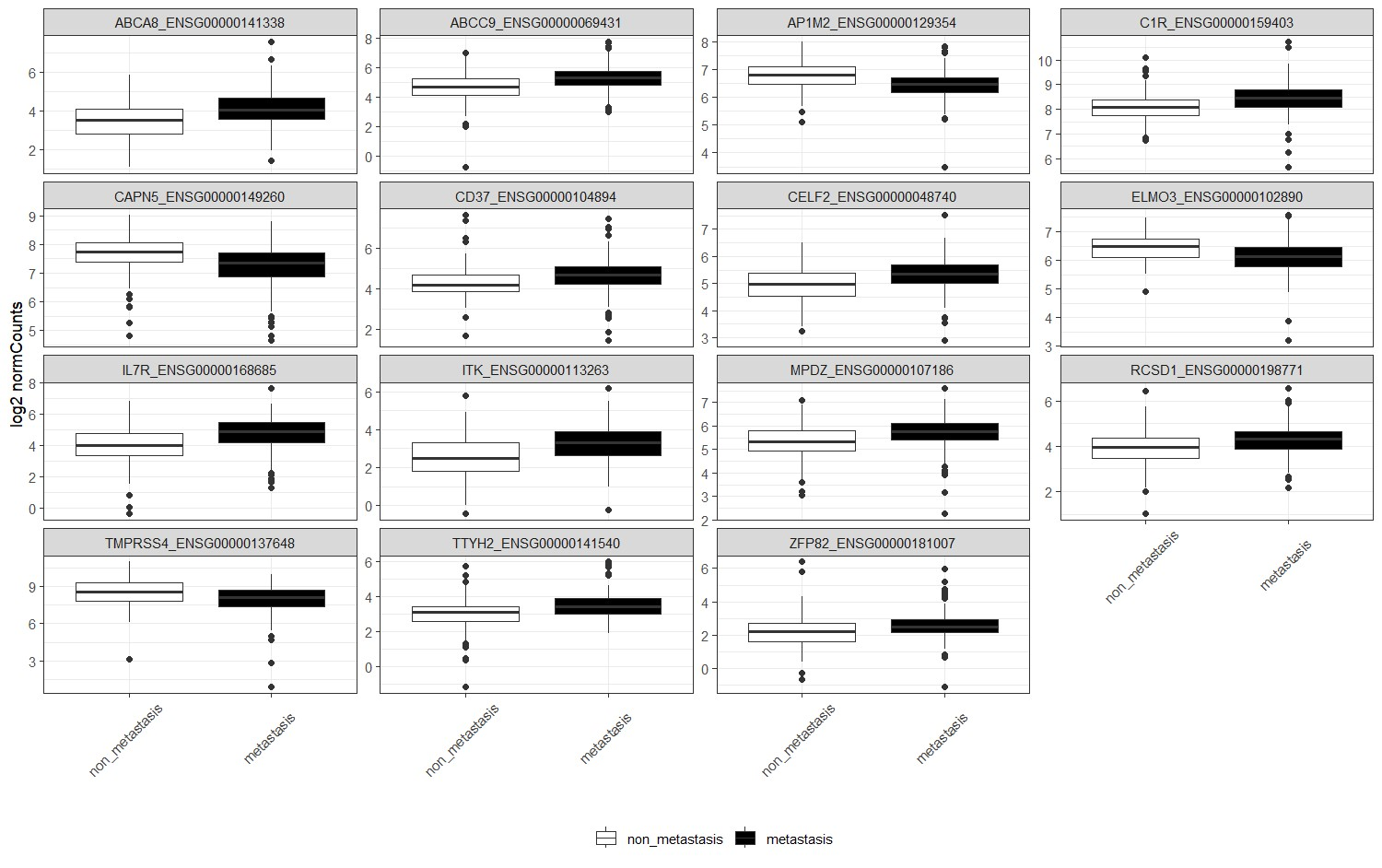


**Figure S4** – boxplots of selected biomarker candidate genes showing gene expression by class

| **No** | **Gene name** | **Protein class** | **Pancreatic cell types enrichment** | **Antibody staining in PDAC samples** |
| --- | --- | --- | --- | --- |
| 1 | C1R | Human disease related genes | Fibroblasts | Mainly low staining and weak intensity |
| 2 | CELF2 | Disease related genes,Human disease related genes | Macrophages | Mostly undetected |
| 3 | ABCC9 | Disease related genes,FDA approved drug targets,Human disease related genes,Metabolic proteins, and Transporters | Endothelial cells | Mainly low staining and weak intensity |
| 4 | ELMO3 | NA | Ductal cells and endothelial cells | Low to medium staining ,Weak to moderate intensity |
| 5 | CD37 | CD markers | Macrophages | Mostly undetected |
| 6 | MPDZ | Disease related genes,Human disease related genes,Plasma proteins | Endothelial cells | Various staining from low to high and strong to weak intensity |
| 7 | ITK | Cancer-related genes, disease related genes,Enzymes,Human disease related genes,Potential drug targets,Human disease related genes | T cells | NA |
| 8 | AP1M2 | NA | Ductal cells and Macrophages | NA |
| 9 | TMPRSS4 | Enzymes,Plasma proteins | Ductal cells | Various staining from low to high and strong to weak intensity |
| 10 | ABCA8 | Metabolic proteins, Transporters | Fibroblasts | Mostly undetected |
| 11 | TTYH2 | Transporters | Endothelial cells | NA |
| 12 | IL7R | Cancer-related genes,CD markers, disease related genes,Human disease related genes | T cells | NA |
| 13 | ZFP82 | Transcription factors | Endothelial cells | Mostly undetected |
| 14 | RCSD1 | NA | Macrophages | Mostly undetected |
| 15 | CAPN5 | Disease related genes,Enzymes,Human disease related genes,Potential drug targets | Fibroblasts and T cells | Various staining from low to high and strong to weak intensity |

**Table 1S**  –Validation in Human Protein Atlas
