# Supplementary material for: Robust and consistent biomarker candidates identification by a machine learning approach applied to pancreatic ductal adenocarcinoma metastasis
Source: BMC Med Inform Decis Mak. 2024 Jun 20;24(Suppl 4):175. doi: 10.1186/s12911-024-02578-0 (PMC11191155; doi:10.1186/s12911-024-02578-0)
Supplement: Supplementary file 2 — Additional file 2. Model evaluation and comparisons. [file 12911_2024_2578_MOESM2_ESM.docx]

**Supplementary Data 2: Model evaluation and comparison**

**Part1: Model evaluation**

The random forest models were evaluated by 12 evaluation metrics which are suitable for evaluating imbalanced data, including Precision, Recall and F1 of the metastasis (class 0), Precision, Recall and F1 of the non-metastasis (class1), Macro-Averaged of Precision, Recall and F1, Receiver Operating Characteristic - Area Under the Curve (ROC_AUC), Precision-Recall - Area Under the Curve (PR_AUC), and Matthews Correlation Coefficient (MCC). The details of each evaluation metric can be found in Table 9S below.

*Table 9S – Evaluation metrics used in this study.*

| **No** | **Metric** | **Calculation** | **Brief Principle** |
| --- | --- | --- | --- |
| 1 | Precision | $\text{Precision}=\frac{\text{TP}}{\text{TP}+\text{FP}}$ | Measures the proportion of true positive predictions  (1). |
| 2 | Recall | $\text{Recall}=\frac{\text{TP}}{\text{TP}+\text{FN}}$ | Measures the proportion of actual positives that are correctly identified(1). |
| 3 | F1 Score | $\text{F1}=2*\frac{\text{Precision*Recall}}{\text{Precision+Recall}}$ | Harmonic mean of Precision and Recall(1). |
| 4 | Macro-Averaged Precision | Calculated for each class separately and then averaged. | Arithmetic mean of the precision values for all classes(1). |
| 5 | Macro-Averaged Recall | Calculated for each class separately and then averaged. | Arithmetic mean of the recall values for all classes (1). |
| 6 | Macro-Averaged F1 Score | Calculated for each class separately and then averaged. | Arithmetic mean of the F1 scores for all classes(1). |
| 7 | Area Under Precision-Recall Curve (PR_AUC) | Computed using the height of the precision by the recall values. | Measures the area under the Precision-Recall curve(2). |
| 8 | Area Under ROC Curve (ROC_AUC) | Computed using the height of the recall (sensitivity) values by the false positive rate. | Measures the area under the Receiver Operating Characteristic curve(ROC) (3). |
| 9 | Matthews Correlation Coefficient (MCC) | MCC =  $\frac{\text{TP}\times\text{TN}-\text{FP}\times\text{FN}}{\sqrt{(\text{TP}+\text{FP})(\text{TP}+\text{FN})(\text{TN}+\text{FP})(\text{TN}+\text{FN})}}$ | Measures the quality of binary and multiclass classifications (4). |

**Part 2: Hyperparameter tuning on the Random Forest (RF)**

The RF model's hyperparameters include *ntree, mtry, splitrule*, and *min.node.size* . The *ntree* parameter determines the number of trees in the forest, *mtry* controls the number of features considered for splitting at each node, *splitrule* sets the rule used for node splitting, and *min.node.size* specifies the minimum number of observations required for a node to split. The configuration of these hyperparameters influences the model's capacity to learn from the data and generalise to unseen data. The details of evaluation metrics and hyperparameters can be found in Table 10S below.

*Table 10S –Summary of the role, meaning, and impact of each hyperparameter on the Random Forest (RF) classifier*

| **Hyperparameter** | **Role** | **Meaning** | **Impact on RF Classifier** |
| --- | --- | --- | --- |
| *ntree* | Determines the number of trees in the forest | The number of decision trees that are included in the random forest | A larger number of trees can increase model performance and computational complexity. Too many trees can lead to overfitting (5). |
| *mtry* | Controls the number of features considered for splitting at each node | The number of predictors sampled at each split when creating each tree | A smaller *mtry* can increase the diversity among the trees and thus the robustness of the model, but it may also increase the bias. A larger *mtry* can decrease the bias and diversity among the trees (5). |
| *splitrule* | Determines the rule used for splitting each node | The function used to measure the quality of a split | Different split rules can lead to different tree structures. For example, the Gini index creates balanced trees, while the information gain creates unbalanced trees (5). |
| *min.node.size* | Sets the minimum number of observations required for a node to split | The minimum number of samples required to be at a leaf node | A smaller *min.node.size* can allow the model to learn finer data details but can also lead to overfitting. A larger *min.node.size* can prevent overfitting but may also prevent the model from learning finer details (5). |

**Part 3 :Model performance comparison between Random Forest and XGBoost model**

Our study employed the Random Forest (RF) model, a widely used statistical learning method in data science. The RF model is particularly effective for variable selection, as it identifies the most pertinent variables to be included in a predictive model. We used the RF model with variable selection algorithms such as Boruta and VarselRF. These RF-based algorithms leverage the capabilities of the RF model for their operations (6). We compared the performance of the RF model with the XGBoost model, both of which are popular decision tree algorithms. The key difference between the two lies in the training of decision trees: XGBoost trains decision trees sequentially, making adjustments based on the prior tree's error, while RF creates decision trees in parallel and independently(7). We built XGBoost models using the same procedures as the RF model. We employed the `train` function from the `caret` R package(8), selecting the method as `xgbTree` (7). Hyperparameter tuning was performed with a range of tune grids, following hyperparameters: `nrounds` (the number of boosting rounds), `max_depth` (the maximum depth of a tree), `eta` (the learning rate), `gamma` (the minimum loss reduction required to make a split), `colsample_bytree` (the fraction of columns to be randomly sampled for each tree), `min_child_weight` (the minimum sum of instance weight needed in a child), and `subsample` (the fraction of observations to be randomly sampled for each tree). We applied the XGBoost model to the training data, performed cross-dataset validation (a model trained by train data and tested by validation data), and validation data. The results are presented in Table S11-S13 below. Briefly, RF model is outperformed XGBoost in almost all evaluation metrics and in three modelling approaches. So, RF model is suitable to use in our analysis.

*Table 11S –Model performances on train data (on the train/test splits) showing average values and 95%CI on the validation data (100 models),* *A:RANDOM FOREST and B:XGBOOST*

| *Metrics* | A:Random forest | | B:XGBoost | |
| --- | --- | --- | --- | --- |
|  | **Model Performance on train data** | | **Model Performance on train data** | |
|  | ***Mean*** | **[95%CI]** | ***Mean*** | **[95%CI]** |
| PC0 | 0.925 | [0.872;0.978] | 0.904 | [0.853;0.955] |
| RC0 | 0.889 | [0.826;0.952] | 0.867 | [0.813;0.921] |
| F10 | 0.904 | [0.845;0.963] | 0.882 | [0.829;0.935] |
| PC1 | 0.804 | [0.725;0.883] | 0.807 | [0.721;0.893] |
| RC1 | 0.847 | [0.775;0.919] | 0.767 | [0.685;0.849] |
| F11 | 0.816 | [0.739;0.893] | 0.778 | [0.691;0.865] |
| MPC | 0.864 | [0.796;0.932] | 0.836 | [0.766;0.906] |
| MRC | 0.868 | [0.800;0.936] | 0.837 | [0.764;0.910] |
| MF1 | 0.860 | [0.791;0.929] | 0.830 | [0.756;0.904] |
| ROC_AUC | 0.945 | [0.899;0.991] | 0.928 | [0.873;0.983] |
| PR_AUC | 0.894 | [0.832;0.956] | 0.860 | [0.784;0.936] |
| MCC | 0.731 | [0.642;0.820] | 0.672 | [0.580;0.764] |

N.B. : PC = Precision , RC =Recall, M = Macro average , PR_AUC=Area under the curve of PR curve, ROC_AUC = Area under the curve of ROC curve, MCC = Matthews correlation coefficient , 0 = metastasis , 1 = non-metastasis, CI = Confidence interval

*Table 12S –RF model performance of cross-dataset validation ( a model trained by train data and tested by validation data) ,* *A:RANDOM FOREST and B:XGBOOST*

| **Metrics** | **A:Cross-dataset Validation performance on RF** | **B: Cross-dataset Validation performance on XGB** |
| --- | --- | --- |
| PC0 | 0.946 | 0.924 |
| RC0 | 0.884 | 0.884 |
| F10 | 0.914 | 0.904 |
| PC1 | 0.692 | 0.673 |
| RC1 | 0.837 | 0.767 |
| F11 | 0.758 | 0.717 |
| MPC | 0.819 | 0.799 |
| MRC | 0.861 | 0.826 |
| MF1 | 0.836 | 0.811 |
| ROC_AUC | 0.839 | 0.780 |
| PR_AUC | 0.938 | 0.927 |
| MCC | 0.678 | 0.624 |

*Table 13S – RF model performance in validation data showing average values and 95%CI on the validation data (100 models),*  *A:RANDOM FOREST and B:XGBOOST*

| *Metrics* | A: Random forest | | B: XGBoost | |
| --- | --- | --- | --- | --- |
|  | **Model Performance in Validation data** | | **Model Performance in Validation data** | |
|  | ***Mean*** | **[95%CI]** | ***Mean*** | **[95%CI]** |
| PC0 | 0.928 | [0.876;0.980] | 0.929 | [0.878;0.980] |
| RC0 | 0.971 | [0.937;1.000] | 0.922 | [0.868;0.976] |
| F10 | 0.947 | [0.902;0.992] | 0.923 | [0.870;0.976] |
| PC1 | 0.911 | [0.854;0.968] | 0.757 | [0.671;0.843] |
| RC1 | 0.740 | [0.652;0.828] | 0.789 | [0.707;0.871] |
| F11 | 0.793 | [0.712;0.874] | 0.748 | [0.661;0.835] |
| MPC | 0.919 | [0.864;0.974] | 0.859 | [0.789;0.929] |
| MRC | 0.855 | [0.785;0.925] | 0.840 | [0.767;0.913] |
| MF1 | 0.870 | [0.803;0.937] | 0.836 | [0.762;0.910] |
| ROC_AUC | 0.941 | [0.894;0.988] | 0.919 | [0.864;0.974] |
| PR_AUC | 0.897 | [0.836;0.958] | 0.827 | [0.751;0.903] |
| MCC | 0.765 | [0.680;0.850] | 0.693 | [0.601;0.785] |

**Part4 : Comparative Analysis of Random Forest Model Performance: 15 final genes and 15 random genes**

We implemented the same analysis approach for validation using 15 randomly selected genes from a pool of 1,023 genes. A Random Forest (RF) model was trained on the train data and subsequently tested using validation data, as depicted in Figure 7S. This process was repeated 100 times to ensure robustness. The performance of the model was then recorded, and the mean and 95% confidence interval were calculated to provide a comprehensive view of the model's performance.


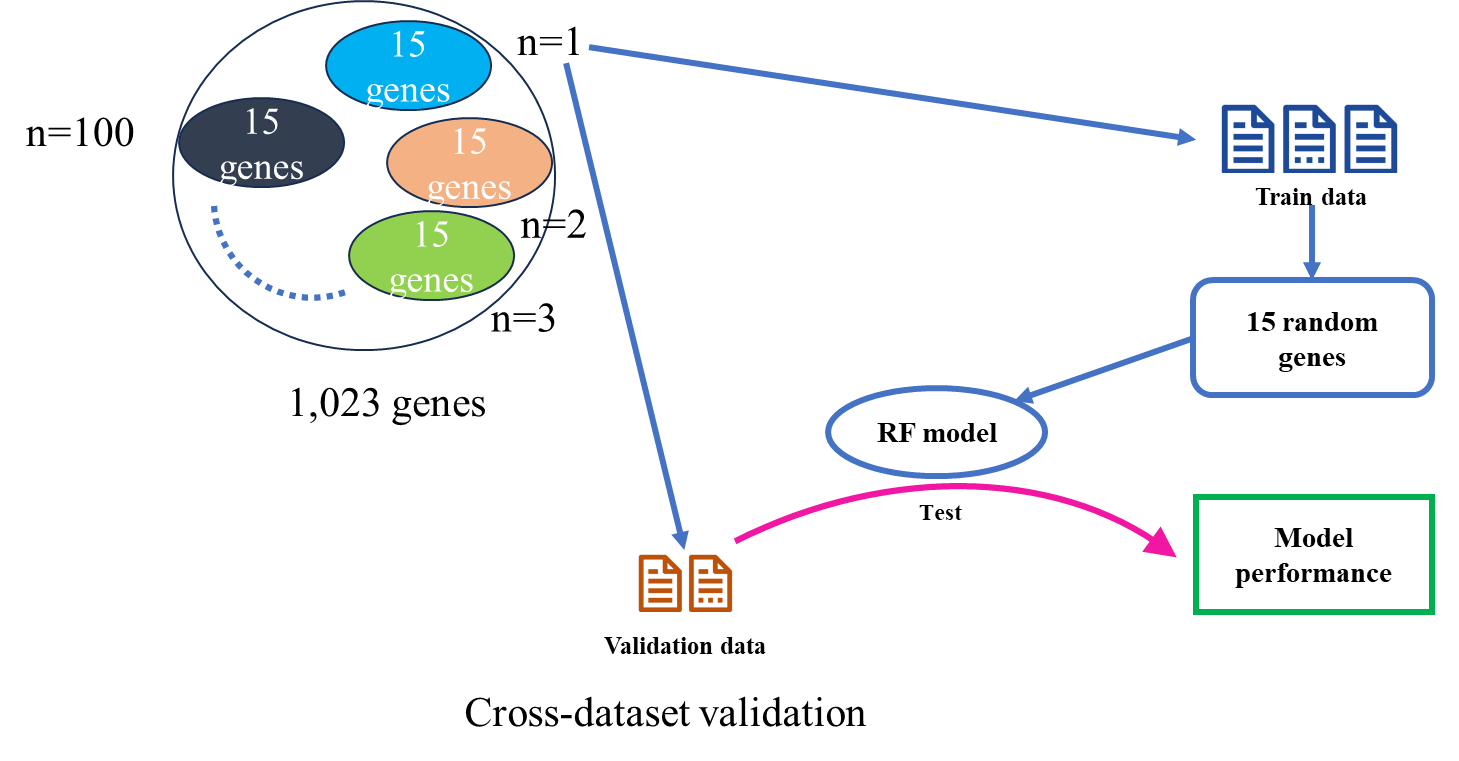


Figure 7S – Analysis workflow of validation performance from 15 random genes (100 models)

The results of RF model performance were illustrated in boxplots (Figure 8S). Clearly, random genes are poorly performed in almost all metrics. In the model performance comparison, the RF model performs significantly better when trained on the 15 final genes compared to the 15 random genes, as shown in Table 14S. These results suggest that the selection of genes significantly impacts the performance of the RF model. The model trained on the final genes consistently outperforms the model trained on the random genes across all metrics, indicating that these genes likely contain more informative variables. This underscores the importance of variable selection in machine learning models for gene expression data.


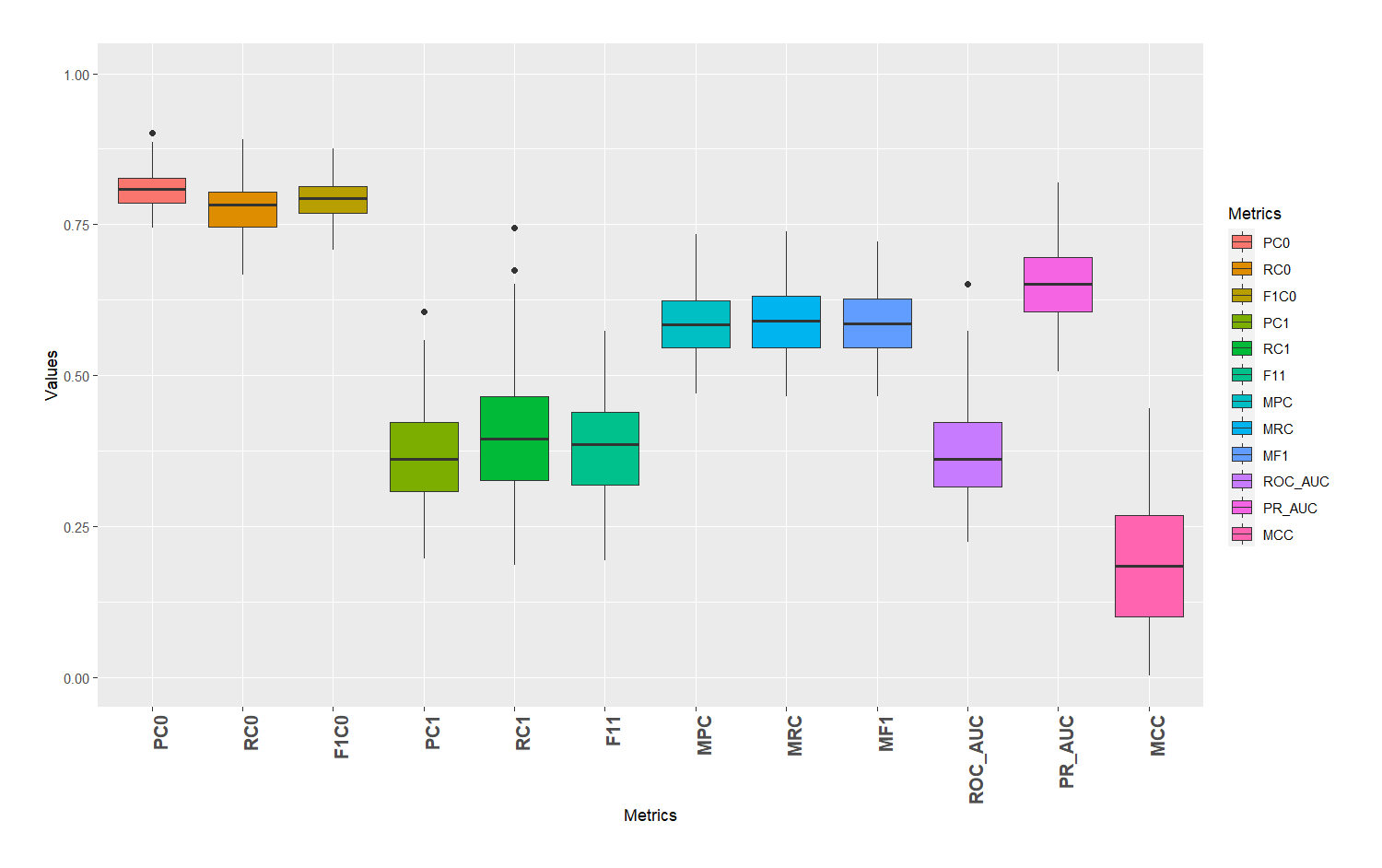
Figure 8S – Boxplot of cross-dataset validation performance from 15 random genes (100 models) showing in each evaluation metrics (PC = Precision, RC =Recall, M = Macro average , PR_AUC=Area under the curve of PR curve, ROC_AUC = Area under the curve of ROC curve, MCC = Matthews correlation coefficient , 0 = metastasis , 1 = non-metastasis)

*Table 14S – Comparison of cross-dataset validation performance in RF models between* *15 final genes(A) and 15 random genes (B) showing average values and 95%CI on the validation data*

| *Metrics* | A: | B: | |
| --- | --- | --- | --- |
|  | **15 final genes**  **(1 model)** | **15 random genes(100 models)** | |
|  |  | ***Mean*** | **[95%CI]** |
| PC0 | 0.946 | 0.809 | [0.730;0.888] |
| RC0 | 0.884 | 0.777 | [0.694;0.860] |
| F10 | 0.914 | 0.792 | [0.711;0.873] |
| PC1 | 0.692 | 0.364 | [0.268;0.460] |
| RC1 | 0.837 | 0.409 | [0.311;0.507] |
| F11 | 0.758 | 0.383 | [0.286;0.480] |
| MPC | 0.819 | 0.586 | [0.488;0.684] |
| MRC | 0.861 | 0.593 | [0.495;0.691] |
| MF1 | 0.836 | 0.587 | [0.489;0.685] |
| ROC_AUC | 0.839 | 0.375 | [0.278;0.472] |
| PR_AUC | 0.938 | 0.652 | [0.557;0.747] |
| MCC | 0.678 | 0.179 | [0.102;0.256] |

**Part 5: Model validation approaches**

We ensure our analysis workflow can avoid the data leakage as the variable selection was performed in only train data. To demonstrate a high degree of confidence in our modelling methodology, we performed internal validation in the training model (Figure 9S-A below) and two validation processes, including cross-dataset (Figure 9S-B) and pseudo-external validation (Figure 9S-C). These three approaches gave excellent results in model performance as shown in the manuscript.


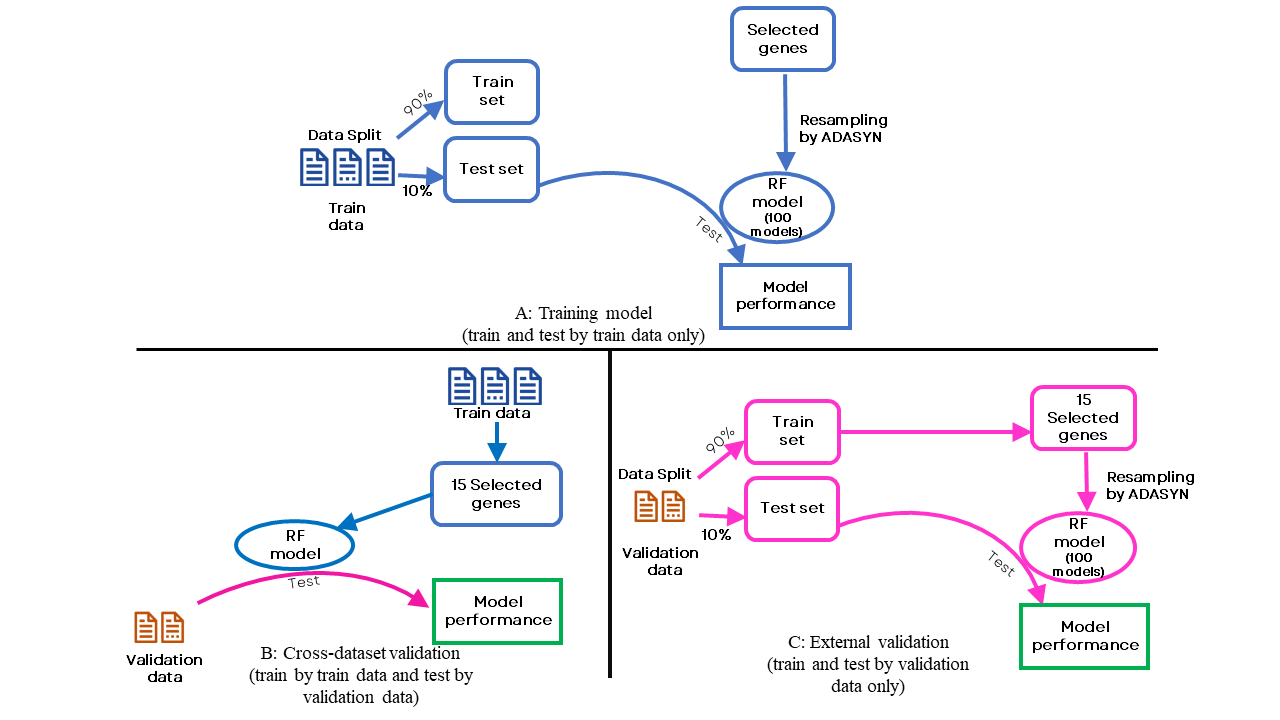


Figure 9S – Model validation approaches implemented in study including A) internal validation , B) Cross-dataset validation , and C) External validation

**References**

1. Sun Y, Wong AKC, Kamel MS. Classification of Imbalanced Data: A Review. International Journal of Pattern Recognition and Artificial Intelligence. 2011;23(04):687-719.

2. Sofaer HR, Hoeting JA, Jarnevich CS, McPherson J. The area under the precision‐recall curve as a performance metric for rare binary events. Methods in Ecology and Evolution. 2019;10(4):565-77.

3. Melo F. Area under the ROC Curve. In: Dubitzky W, Wolkenhauer O, Cho K-H, Yokota H, editors. Encyclopedia of Systems Biology. New York, NY: Springer New York; 2013. p. 38-9.

4. Chicco D, Jurman G. The advantages of the Matthews correlation coefficient (MCC) over F1 score and accuracy in binary classification evaluation. BMC Genomics. 2020;21(1):6.

5. Bartz-Beielstein T, Chandrasekaran S, Rehbach F, Zaefferer M. Case Study I: Tuning Random Forest (Ranger). In: Bartz E, Bartz-Beielstein T, Zaefferer M, Mersmann O, editors. Hyperparameter Tuning for Machine and Deep Learning with R: A Practical Guide. Singapore: Springer Nature Singapore; 2023. p. 187-220.

6. Iranzad R, Liu X. A review of random forest-based feature selection methods for data science education and applications. International Journal of Data Science and Analytics. 2024.

7. Chen T, Guestrin C. XGBoost: A scalable tree boosting system. Proceedings of the ACM SIGKDD International Conference on Knowledge Discovery and Data Mining. 2016;13-17-August-2016:785-94.

8. Kuhn M. Building predictive models in R using the caret package. Journal of Statistical Software. 2008;28(5):1-26.
