# Supplementary material for: Robust and consistent biomarker candidates identification by a machine learning approach applied to pancreatic ductal adenocarcinoma metastasis
Source: BMC Med Inform Decis Mak. 2024 Jun 20;24(Suppl 4):175. doi: 10.1186/s12911-024-02578-0 (PMC11191155; doi:10.1186/s12911-024-02578-0)
Supplement: Supplementary file 4 — Additional file 4. Results from GeneMANIA and QIAGEN IPA. [file 12911_2024_2578_MOESM4_ESM.docx]

**Biological Contextualisation**

**GeneMANIA**


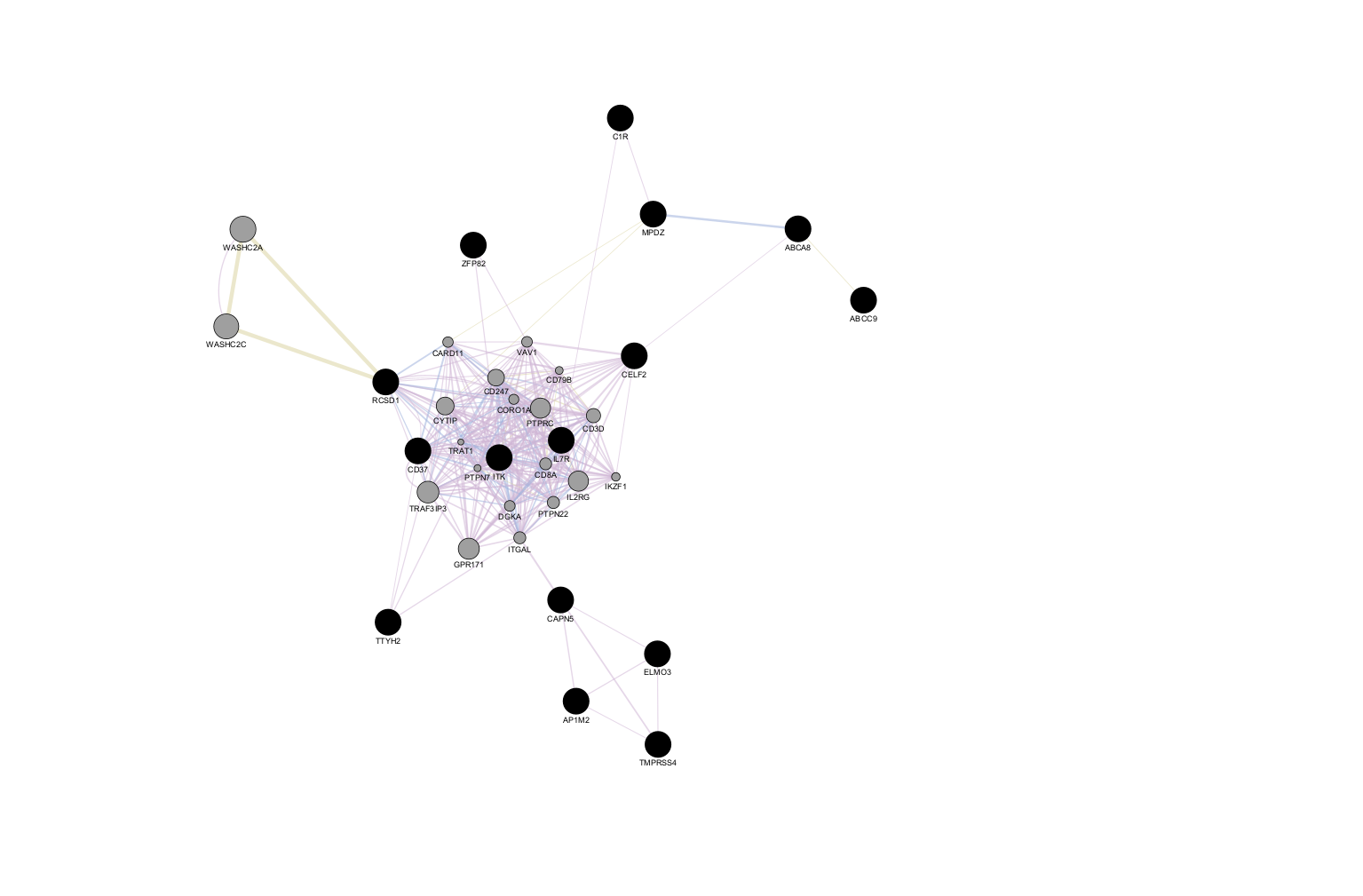


Figure 5S – Gene network of 15 final genes and top 20 relevant genes by GeneMANIA

**Table 2S** – **Significant GO biological processes from GeneMANIA**

| GO id | Description | q-value | Occurrences in Sample | Occurrences in Genome |
| --- | --- | --- | --- | --- |
| GO:0050851 | antigen receptor-mediated signalling pathway | 8.17E-05 | 8 | 222 |
| GO:0098802 | plasma membrane signalling receptor complex | 0.003563728 | 6 | 161 |
| GO:0043235 | receptor complex | 0.01220029 | 6 | 217 |
| GO:0050854 | regulation of antigen receptor-mediated signalling pathway | 0.01220029 | 4 | 54 |
| GO:0030098 | lymphocyte differentiation | 0.013913889 | 6 | 238 |
| GO:0038093 | Fc receptor signalling pathway | 0.040094436 | 5 | 171 |
| GO:0006909 | phagocytosis | 0.059131822 | 5 | 200 |
| GO:0050856 | regulation of T cell receptor signalling pathway | 0.059131822 | 3 | 31 |
| GO:0050852 | T cell receptor signalling pathway | 0.059131822 | 3 | 33 |

**Table 3S**  – **Node information of gene network from GeneMANIA**

| Gene | Score | Description |
| --- | --- | --- |
| ZFP82 |  | ZFP82 zinc finger protein |
| ABCC9 |  | ATP binding cassette subfamily C member 9 |
| RCSD1 |  | RCSD domain containing 1 |
| TTYH2 |  | tweety family member 2 |
| ABCA8 |  | ATP binding cassette subfamily A member 8 |
| MPDZ |  | multiple PDZ domain crumbs cell polarity complex component |
| ELMO3 |  | engulfment and cell motility 3 |
| CELF2 |  | CUGBP Elav-like family member 2 |
| AP1M2 |  | adaptor related protein complex 1 subunit mu 2 |
| TMPRSS4 |  | transmembrane serine protease 4 |
| CAPN5 |  | calpain 5 |
| ITK |  | IL2 inducible T cell kinase |
| CD37 |  | CD37 molecule |
| IL7R |  | interleukin 7 receptor |
| C1R |  | complement C1r |
| WASHC2A | 0.57 | WASH complex subunit 2A |
| WASHC2C | 0.57 | WASH complex subunit 2C |
| TRAF3IP3 | 0.54 | TRAF3 interacting protein 3 |
| GPR171 | 0.54 | G protein-coupled receptor 171 |
| IL2RG | 0.53 | interleukin 2 receptor subunit gamma |
| PTPRC | 0.53 | protein tyrosine phosphatase receptor type C |
| CYTIP | 0.52 | cytohesin 1 interacting protein |
| CD247 | 0.51 | CD247 molecule |
| CD3D | 0.49 | CD3d molecule |
| PTPN22 | 0.48 | protein tyrosine phosphatase non-receptor type 22 |
| ITGAL | 0.48 | integrin subunit alpha L |
| CD8A | 0.47 | CD8a molecule |
| DGKA | 0.47 | diacylglycerol kinase alpha |
| VAV1 | 0.47 | vav guanine nucleotide exchange factor 1 |
| CARD11 | 0.46 | caspase recruitment domain family member 11 |
| CORO1A | 0.46 | coronin 1A |
| IKZF1 | 0.45 | IKAROS family zinc finger 1 |
| CD79B | 0.45 | CD79b molecule |
| PTPN7 | 0.44 | protein tyrosine phosphatase non-receptor type 7 |
| TRAT1 | 0.43 | T cell receptor associated transmembrane adaptor 1 |

**Table 4S**  – **Edge information of gene network from GeneMANIA**

| Network | Weight | Title |
| --- | --- | --- |
| **Co-expression** | **85.69%** |  |
| Arijs-Rutgeerts-2009 | 14.07 | Mucosal gene expression of antimicrobial peptides in inflammatory bowel disease before and after first infliximab treatment. |
| Chen-Brown-2002 | 13 | Gene expression patterns in human liver cancers. |
| Wang-Maris-2006 | 11.76 | Integrative genomics identifies distinct molecular classes of neuroblastoma and shows that multiple genes are targeted by regional alterations in DNA copy number. |
| Innocenti-Brown-2011 | 9.67 | Identification, replication, and functional fine-mapping of expression quantitative trait loci in primary human liver tissue. |
| Roth-Zlotnik-2006 | 9.67 | Gene expression analyses reveal molecular relationships among 20 regions of the human CNS. |
| Mallon-McKay-2013 | 7.22 | StemCellDB: the human pluripotent stem cell database at the National Institutes of Health. |
| Dobbin-Giordano-2005 | 5.27 | Interlaboratory comparability study of cancer gene expression analysis using oligonucleotide microarrays. |
| Bild-Nevins-2006 B | 4.97 | Oncogenic pathway signatures in human cancers as a guide to targeted therapies. |
| Perou-Botstein-2000 | 4.67 | Molecular portraits of human breast tumours. |
| Rosenwald-Staudt-2001 | 3.51 | Relation of gene expression phenotype to immunoglobulin mutation genotype in B cell chronic lymphocytic leukemia. |
| Wang-Cheung-2015 | 1.87 | Genetic variation in insulin-induced kinase signaling. |
| **Co-localization** | **12.53%** |  |
| Johnson-Shoemaker-2003 | 12.53 | Genome-wide survey of human alternative pre-mRNA splicing with exon junction microarrays. |
| **Shared protein domains** | **1.78%** |  |
| INTERPRO | 1.19 |  |
| PFAM | 0.59 |  |

**QIAGEN IPA**

**Top conical pathways in variable set 1-3**

**Table 5S**  – **Set 1, final genes from ML analysis (Final)**


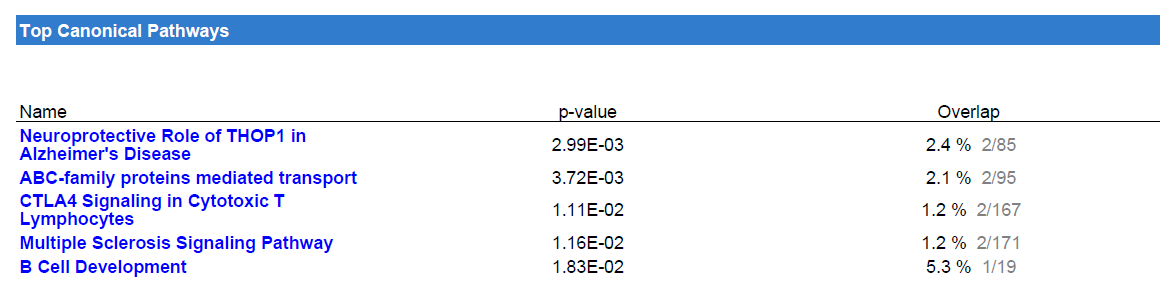


**Table 6S**  – **Set 2 , differentially secreted proteins(DE)**


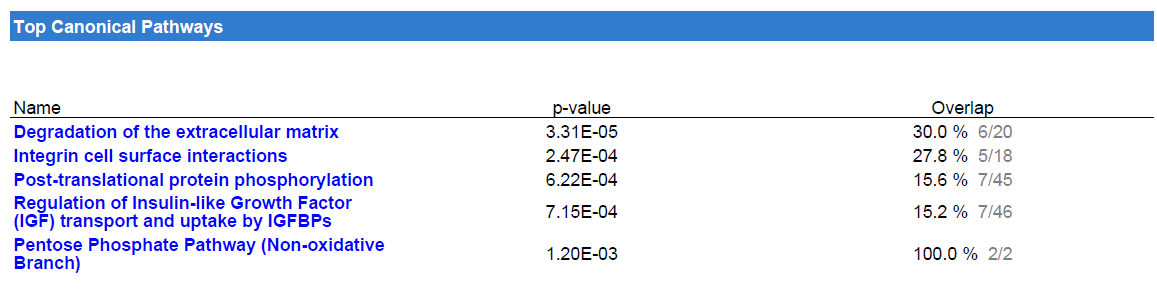


**Table 7S**  – **Set 3,exclusively secreted proteins (EXCLU)**


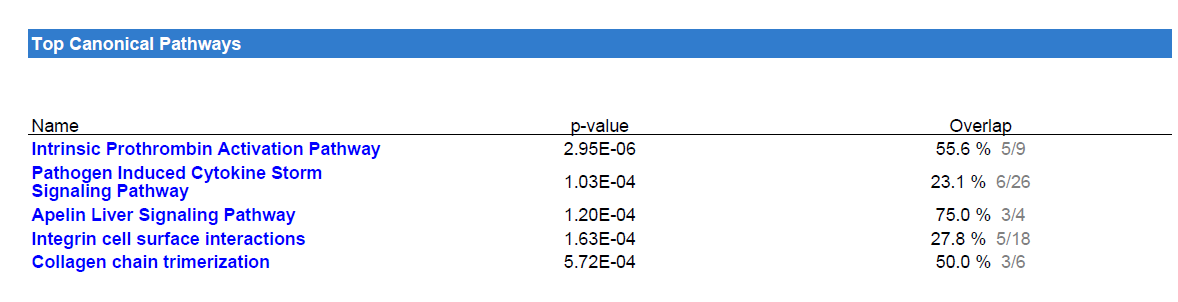


**Table 8S**  – **Networks of Top Diseases and Functions enriched from set 1- 3**

| ID | Analysis | Score | Focus Molecules | Top Diseases and Functions |
| --- | --- | --- | --- | --- |
| 1 | Final only | 28 | 11 | [Cancer, Cellular Development, Organismal Injury and Abnormalities] |
| 1 | DE only | 41 | 18 | [Cellular Movement, Hematological System Development and Function, Immune Cell Trafficking] |
| 1 | EXCLU only | 34 | 15 | [Cellular Movement, Hematological System Development and Function, Tissue Development] |
| 2 | Final only | 3 | 1 | [Cell Morphology, Cellular Assembly and Organization, DNA Replication, Recombination, and Repair] |
| 2 | DE only | 24 | 12 | [Cell Morphology, Cellular Assembly and Organization, Cellular Movement] |
| 2 | EXCLU only | 18 | 9 | [Cell Death and Survival, Lipid Metabolism, Organismal Injury and Abnormalities] |
| 3 | Final only | 3 | 1 | [Auditory and Vestibular System Development and Function, Cell Cycle, Cellular Development] |
| 3 | DE only | 8 | 5 | [Cardiovascular Disease, Connective Tissue Development and Function, Organismal Injury and Abnormalities] |
| 3 | EXCLU only | 9 | 5 | [Cell Death and Survival, Inflammatory Response, Organismal Injury and Abnormalities] |
| 4 | DE only | 2 | 1 | [Hereditary Disorder, Neurological Disease, Organismal Injury and Abnormalities] |
| 4 | EXCLU only | 2 | 1 | [Hereditary Disorder, Organismal Injury and Abnormalities, Reproductive System Disease] |


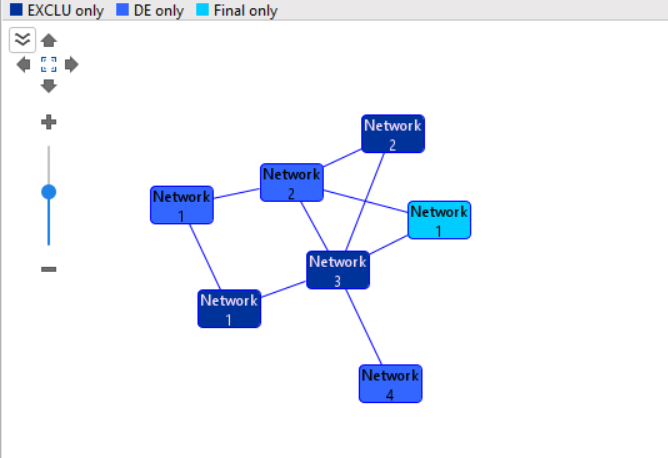


Figure 6S – Overlapping network from set 1- 3 : Network 1 of set1 is overlapping with network 2 of set2(DE) and network 3 of set3 (EXCLU)
